# Exploring the Genome and Phenotype of Multi-Drug Resistant *Klebsiella pneumoniae* of Clinical Origin

**DOI:** 10.3389/fmicb.2017.01913

**Published:** 2017-10-23

**Authors:** João Anes, Daniel Hurley, Marta Martins, Séamus Fanning

**Affiliations:** ^1^UCD-Centre for Food Safety, School of Public Health, Physiotherapy and Sports Science, University College Dublin, Dublin, Ireland; ^2^Institute for Global Food Security, Queen's University Belfast, Belfast, United Kingdom

**Keywords:** antimicrobial resistance, biofilm formation, *Klebsiella pneumonia*, MDR phenotypes, whole genome analysis

## Abstract

*Klebsiella pneumoniae* is an important nosocomial pathogen with an extraordinary resistant phenotype due to a combination of acquired resistant-elements and efflux mechanisms. In this study a detailed molecular characterization of 11 *K. pneumoniae* isolates of clinical origin was carried out. Eleven clinical isolates were tested for their susceptibilities, by disk diffusion and broth microdilution and interpreted according to CLSI guidelines. Efflux activity was determined by measuring the extrusion of ethidium bromide and biofilm formation was assessed following static growth in Müeller-Hinton and minimal media M9 broths at two temperatures and time points. Template DNA from all 11 isolates was extracted and sequenced. The study collection was found to be resistant to several (extended-spectrum beta-lactam) ESBL-type compounds along with several (fluoro)quinolones (FQ). Resistance to tetracycline accounted for 55% of the study collection (*n* = 6) and three of the 11 isolates were resistance to carbapenems. Genotyping identified *bla*_CTX-*M*-15_ (82%), *bla*_SHV-12_ (55%), and *bla*_TEM-1*B*_ (45%) ESBL encoding genes and FQ resistance was associated the presence of the *oqxAB* operon, identified in 10 of the 11 isolates and *qnrB* gene in one isolate. The polymorphisms detected in the quinolone resistance-determining regions (QRDRs) were associated with isolates of the clonal group CG15. Sequence types (ST) identified were representative of previously described clonal groups including CG258 (*n* = 7), CG15 (*n* = 3), and CG147 (*n* = 1). Plasmid replicon type databases were queried indicating the presence of IncFII and IncFIB replicon types in the majority of the isolates (91%), followed by IncFIA (45%), and IncR (45%). Two of the 11 isolates were found positive for yersiniabactin siderophore-encoding genes. No differences in the ability to efflux ethidium bromide were identified. Biofilm formation was stronger when the isolates were grown under stressed conditions at 37°C for a period up to 96 h. These data confirm the fact that well-recognized clonal groups of *K. pneumoniae* of importance to human health carries a diverse repertoire of antimicrobial resistance determinants, particularly related to critically important drugs in the ESBL and FQ classes. The capacity of most isolates to form strong biofilms, when stressed under laboratory-simulated conditions, supports the risk to human health associated with nosocomial infections deriving from indwelling medical devices.

## Introduction

*Klebsiella pneumoniae* is a non-motile, lactose-fermenting, capsulated bacillus belonging to the Enterobacteriaceae family. It can be found in the soil, water, plants (Seidler et al., 1975; Bagley, 1985; Barati et al., 2016), and in the gastrointestinal tracts of animals and humans (Kim et al., 2005). For the most part, carriage of this organism in an immune-competent individual is regarded as benign. Nonetheless, this bacterium is increasingly being associated with cases of community-, and hospital-acquired infections. Hospital-acquired (HA) infections epidemiologically linked to *K. pneumoniae* often present as bloodstream infections (BSI), along with infections of the abdominal cavity, surgical sites, soft tissue(es), meningitis, and also respiratory and urinary tract infection (UTI), particularly in immunocompromised patients, the elderly and neonates (Podschun and Ullmann, 1998; Lee and Burgess, 2012; Janda, 2015). In contrast, community-acquired (CA) infections are recognized by the expression of a hypermuicoid phenotype associated with the capsule producing serotypes K1 and K2 together with various siderophores (Shon et al., 2013). The latter can infect healthy individuals and this can lead to pyogenic liver abscesses (PLA), Lemierre's syndrome, antibiotic-associated haemorrhagic colitis with the potential to disseminate to other parts of the body resulting in endophtalmitis and meningitis with mortality rates ranging from 5 up to 30% being reported (Janda, 2015).

Until recently *K. pneumoniae* was mainly considered as a nosocomial pathogen. However, detections of *Klebsiella* species cultured from various retail meat products suggests that this pathogen may be disseminating by hitherto unrecognized routes such as the modern food chain (Davis et al., 2015). The paucity of surveillance data along the food chain is likely to have masked this observation and should be re-evaluated for its risk to public health. Further, 39% of the BSI infections reported in Ireland are linked to gastrointestinal infections (Brady et al., 2016).

The extensive use of antimicrobial compounds both in hospitalized patients and in agriculture has led to the development of multi-drug resistance (MDR) among several bacterial genera, including *Klebsiella* species. With the well-documented dissemination of extended-spectrum beta-lactamase- (ESBLs) and carbapenemase-encoding genes in *Klebsiella* this pathogen is considered one of the leading causes of nosocomial infections thereby narrowing the chemotherapeutic options for treatment (Arnold et al., 2011). Many of the resistance- and virulence-encoding genes can be mapped to mobile genetic elements (MGE), such as plasmids (Ramirez et al., 2014). Thus, *Klebsiella* species is recognized as a reservoir for MDR gene containing plasmids a feature that has contributed for the spread of ESBLs and carbapenemases among Enterobacteriaceae (Tzouvelekis et al., 2012). This development is now viewed as serious challenge to public health (Centres for Desease Control and Prevention, 2013; World Health Organization, 2014).

*Klebsiella* species have the ability to form biofilms especially on abiotic surfaces such as on plastic, used in indwelling instruments for instance catheters and endotracheal tubing. This ability is a feature that contributes to its role in urinary tract infections (UTIs; Arana et al., 2016). These plastic surfaces provide an inert matrix suitable for bacterial attachment through their type-1 and -3 fimbriae adhesins (Struve et al., 2008, 2009; Murphy et al., 2013). Biofilms consist of aggregated bacterial cells confined within a matrix of polysaccharides and DNA, and capable of resisting the host's defense mechanisms and exposure to antibiotics. These phenotypes arise following alterations in the bacterium related to permeability changes, efficient diffusion of molecules within the matrix and biofilm senescence (Stewart, 2003, 2015). Resistance to antimicrobial agents can be extended up to 1,000-fold when compared with the measurements made with planktonic cells (Ceri et al., 1999; Singla et al., 2013).

In this study, eleven *K. pneumoniae* isolates of clinical origin were characterized. Their susceptibility to a panel of antimicrobial agents was measured along with their membrane-mediated efflux activity and abilities to form biofilms under defined laboratory-induced conditions. WGS analysis identified the clonal groups in each case and provided a catalog of resistance-related genotypes and virulence factors, thereby enabling a comparison with globally reported members of this bacterial species.

## Materials and methods

### Bacterial culture media and other chemicals

Müeller-Hinton (MH) broth and Müeller-Hinton agar (MHA), phosphate buffered saline (PBS), and all powder-based antimicrobial compounds and reagents, with exception of moxifloxacin, were obtained from Sigma-Aldrich (Ireland). Moxifloxacin was purchased from Santa Cruz Biotechnology (Spain). All the diffusion disk antibiotics were purchased from Oxoid (Thermo Fisher Scientific).

### Bacterial study isolates

Eleven *K. pneumoniae* clinical isolates obtained in 2008 from a major tertiary teaching hospital, in Dublin were used in this study. The control isolate *Escherichia coli* ATCC™25922 was used in antibiotic susceptibility tests and the isolate *E. coli* ATCC™8739 was used for MALDI-TOF internal control. Similarly, *K. pneumoniae* MGH 78578 (ATCC™700721) also included as control for MDR *K. pneumoniae*. *Salmonella* Typhimurium ATCC™14028 was used as positive control for biofilm formation and RADR morphotype.

### Growth curves

Bacterial growth was measured in MH broth. Briefly, overnight cultures of *K. pneumoniae* were washed twice with PBS and diluted in MH broth to an OD_600_ 0.005. Two hundred microliters of each diluted isolate was pipetted into a 96-well plate and absorbance was measured over a 24 h period, using a Multiskan™ FC Microplate Photometer (Thermo Scientific, IE) at 620 nm, incubated at 37°C. Each isolate was cultured in triplicate with five technical replicates for this assay.

### Antibiotic susceptibility test (AST)

The antibiotic susceptibility of each *Klebsiella* isolate was determined by disk diffusion (Bauer et al., 1966). In brief, bacterial cultures were grown overnight and diluted in sterile PBS to 0.5 McFarland standard and spread, using a cotton-tipped bud across dried MHA-plates. Disks containing amikacin (AK—30 μg), aztreonam (ATM—30 μg), cefepime (FEP—30 μg), cefotaxime (CTX—5 μg), ceftazidime (CAZ—10 μg), chloramphenicol (C—30 μg), ciprofloxacin (CIP—5 μg), doripenem (DOR—10 μg), doxycycline (DO—30 μg), ertapenem (ETP—10 μg), gentamicin (CN—10 μg), imipenem (IPM—10 μg), levofloxacin (LEV—5 μg), meropenem (MEM—10 μg), minocycline (MH—30 μg), moxifloxacin (MXF—5 μg), nalidixic acid (NA—30 μg), norfloxacin (NOR—10 μg), penicillin (P—10 iu), tetracycline (TET—30 μg), ticarcillin (TIC—75 μg), ticarcillin-clavulanic acid (TIM—85 μg), tigecyline (TGC—15 μg), and trimethoprim-sulfamethoxazole (SXT—25 μg) were included. Inoculated plates, containing the disks above were incubated at 37°C and any zone of inhibition formed thereafter, was measured after 16–18 h. Results were interpreted as susceptible, intermediate or resistant according to breakpoints as defined by CLSI, M100-S23 document (CLSI, 2013). In the case of moxifloxacin and tigecycline the breakpoints used were those from The European Committee on Antimicrobial Susceptibility Testing—EUCAST (2016). This assay was performed in triplicate for each clinical isolate.

### Minimum inhibitory concentration (MIC) and minimum bactericidal concentration (MBC)

MIC values for cefotaxime (CTX), chloramphenicol (CHL), ciprofloxacin (CIP), colistin (CT), gentamicin (CN), imipenem (IMP), kanamycin (KM), moxifloxacin (MXF), nalidixic acid (NAL), polymyxin B (PB), tetracycline (TET) and Benzalkonium chloride (BK) were measured by two-fold broth microdilution in a 96-well microtiter plates. Briefly, overnight cultures were diluted in sterilized PBS to ~10^5^ CFU/mL. Aliquots of 5 μL were then transferred to separate wells in a 96-well plate that contained 100 μL of each compound at varying concentrations prepared from two-fold serial dilutions in MH broth. The concentration ranges used were 1–512 μg/mL, with the exception of moxifloxacin wherein the concentration ranges were 0.25–128 μg/mL. These plates were incubated at 37°C and the MIC values recorded after 16–18 h. Susceptibility or resistance was determined with reference to the CLSI breakpoints (M100-S23). In the case of colistin, polymyxin B, and moxifloxacin the breakpoints used were those specified by The European Committee on Antimicrobial Susceptibility Testing—EUCAST (2016).

Determination of the MBC values for all antimicrobial agents tested above was performed in MH broth media. Five microliters were transferred from the MICs 96-well plates (above) and re-inoculated into fresh sterile 96-well plates containing MH broth, without any of the antimicrobial compounds. Plates were incubated at 37°C and the MBC results recorded after 16–18 h. All assays were carried out three times for each antibiotic.

### Genomic and plasmid DNA purification

Genomic DNA was purified from overnight cultures using the UltraClean® Microbial DNA Isolation Kit (MoBio Laboratories, CA) according to manufacturer's instructions. Small plasmid DNA (sizes < 30-kbp) extraction was carried out using the High Pure Plasmid Isolation Kit (Roche, SH). *E. coli* 39R 861, and *E. coli* V517 were used as molecular weight controls for plasmid sizing (Wang et al., 2013). Purified DNA was resolved in a 0.8% [w/v] agarose gel, stained with SYBR green.

Large plasmid extraction was carried out using S1-nuclease (Promega, Madison, WI, USA) followed by pulsed-field gel electrophoresis (PFGE; Barton et al., 1995). *E. coli* 39R 861 and *E. coli* V517 were included as controls. Briefly, the procedure included a lysis step for the bacterial cells, previously embedded in agarose plugs followed by digestion with 8 U S1-nuclease at 37°C for 45 min. Finally, each plasmid sample was resolved by PFGE in a Chef-Mapper® XA System (Bio-Rad, USA) at 14°C, with a switch time between 1 and 12 s, at 6 V/cm on a 120° angle in 0.5 × TBE buffer for 18 h in a 0.8% [w/v] agarose gel and stained with SYBR green. The approximate molecular mass of plasmids was determined by comparing band molecular sizes with the two control isolates and by using *Salmonella* Braenderup H9812 digested by XbaI (Wang et al., 2013).

### Biolog identification

Isolate identification was carried out based on the phenotypic growth pattern for different carbon sources using the GEN III MicroPlate™ platform (Biolog Inc., CA, USA). Three single colonies were collected from an overnight culture in MH-agar and mixed with inoculating fluid B (IF-B) prior to being adjusted to a transmittance of 90–98% using a turbidimeter, as specified in the user guide. For each bacterial isolate, 100 μL of the cell suspension was inoculated into each well of the MicroPlates and incubated at 37°C for a period of 20 h. Results were interpreted using the OmniLog® identification system's software (GEN III database, version 5.2.1).

### MALDI-TOF identification

Bacterial isolate identification was further confirmed with a Matrix Assisted Laser Desorption Ionization Time-of-Flight (MALDI-TOF) system based on the VITEK® MS platform (bioMérieux, Marcy l-Etoile, France). Half of a single colony was picked from an overnight culture in MH agar using a 1 μL sterile loop and placed on the target slide. When dry, the spot was covered with 1 μL α-cyano-4-hydrocinnamic acid (CHCA) matrix (bioMérieux, Marcy l-Etoile, France). *E. coli* ATCC™8739 was used as an internal control. Results were analyzed using Myla®, software database for microbial identification.

### Quantitative assessment of efflux by fluorometry using ethidium bromide (EtdBr)

In order to quantitatively assess the efflux ability of the clinical isolates, ethidium bromide was used as described previously (Paixão et al., 2009) with several modifications. Briefly, *K. pneumoniae* overnight cultures were grown in MH broth until mid-log phase. Bacterial cells were washed twice with PBS and OD_600 nm_ readjusted to 0.6. Cells were incubated in PBS in the presence of EtdBr (50 μM) and CCCP (100 μM) at 25°C for 1 h. After that time, cells were washed with PBS, centrifuged and the contents re-suspended in (i) PBS and (ii) PBS with 50 μM CCCP. The cells were immediately pipetted into a 96-well plate and ethidium bromide fluorescence was measured in a Fluoroskan Ascent FL (Thermo Scientfic, IE) with an excitation and emission wavelengths of 518- and 606-nm, respectively. After 3 min, glucose was added to one of the well containing PBS to a final concentration of 50 mM. Fluorescence emitted was acquired in cycles of 60 s, during 50 min at 37°C. Each bacterial isolate was tested in triplicate for this assay. Results were averaged and normalized based on the fluorescence collected from the respective samples in PBS. Finally, results were plotted in terms of relative fluorescence.

### Biofilm formation assay

Biofilm formation was examined under defined growth conditions that experimentally approximate different stress conditions. M9 minimal media (containing NH_4_Cl [1.9 mM], Na_2_HPO_4_ [42.3 mM], KH_2_PO_4_ [22 mM], NaCl [8.56 mM], MgSO_4_ [2 mM], CaCl_2_ [0.1 mM], and glucose [0.1% w/v]) and MH broth were used for this purpose to enable an assessment of biofilm formation capacity, when cultures were incubated at 25 and 37°C respectively for periods ranging from 8 to 96 h. Initially, overnight cultures were adjusted to OD_600_ 0.3 with fresh media, and 200 μL of this bacterial cell suspension was dispensed across a 96-well microtiter plate. Plates were incubated statically for 8–96 h under the same conditions as described above. *Salmonella* Typhimurium ATCC™14028, a proven strong biofilm forming isolate, was used as a positive control (Finn et al., 2013).

Crystal violet was used to detect total biofilm biomass formed (Stepanović et al., 2007). Briefly the 96-well plate containing culture medium was discarded after growth and the plate was washed with PBS. Crystal violet (0.4% [v/v]) was added and incubated at room temperature for 15 min. The plate was then washed again with PBS and dried in a sterile air flow hood for 20 min. Once dried, 200 μL 33% [v/v] acetic acid was added to each well and the absorbance was recorded in a Multiskan™ FC Microplate Photometer (Thermo Scientific, Dublin, Ireland) at 570 nm. This assay was performed in triplicate for each isolate with 5 technical replicates for each biological replicate.

### Congo red and calcofluor staining

Expression of curli fimbriae was assessed by examining colony morphology of *K. pneumoniae* when grown on Congo red agar. Congo red agar was prepared as described by Römling and Rohde (1999). In brief, MH agar was supplemented with 40 μg/mL Congo red (Sigma-Aldrich, Ireland) and 20 μg/mL Coomassie brilliant blue R-250 (Thermo Fisher Scientific, Waltham, MA). Plates were then inoculated with 3 μL of each overnight bacterial culture grown in MH broth and then incubated for 72 h at 25 and 37°C after which colony morphology was inspected and recorded. *Salmonella* Typhimurium ATCC™14028 was included as a control for a RDAR phenotype (Römling and Rohde, 1999; Finn et al., 2013).

Calcofluor agar was used to assess these isolates for their ability to produce cellulose (Zogaj et al., 2001). In this case, the plates were prepared by supplementing MH agar with 40 μg/mL calcofluor fluorescent brightener 28 (Sigma-Aldrich). The calcofluor agar plates were inoculated with 3 μL of overnight culture and incubated for 72 h at 25 and 37°C. *Salmonella* Typhimurium ATCC™14028 was included as a control. The colony morphology was observed and recorded under a 366-nm UV light source to detect the binding of calcofluor to the cellulose produced. Both assays were carried out in triplicate for each isolate.

### Pellicle formation

To examine pellicle formation, 50 μL from an overnight in MH broth was used to inoculate 5 mL MH broth, which was then incubated statically for 96 h at 25 and 37°C after which it was then inspected for the formation of a pellicle at the air-broth interface (Finn et al., 2013). The assay was repeated twice and results were registered as present or absent.

### Bacterial whole genome sequencing

Genomic libraries were prepared commercially (Novogene, Beijing, China) using the Illumina TruSeq library preparation kit for each of the 11 *K. pneumoniae* and sequenced on the HiSeq platform (Illumina). The quality of the reads was assessed using FastQC (version 0.11.5; Marçais and Kingsford, 2011). Error correction was performed using BFC (version r181; Li, 2015). A relaxed quality trim was performed using Trimmomatic (version 0.36) before the genome was *de novo* assembled using SPAdes (version 3.9.1; Bankevich et al., 2012; Bolger et al., 2014). The quality of the subsequent assemblies was assessed using Bandage (version 0.8.1) and QUAST (version 4.3; Gurevich et al., 2013; Wick et al., 2015). Assemblies were annotated using Prokka (version 1.11; Seemann, 2014).

Antimicrobial resistance genes were identified using Resfinder 2.1 (version 2016-11-02; https://cge.cbs.dtu.dk/services/ResFinder/; Zankari et al., 2012). Plasmid replicon typing was performed using PlasmidFinder 1.3 (https://cge.cbs.dtu.dk//services/PlasmidFinder/; Carattoli et al., 2014). Antibacterial biocide and metal resistance genes were using BacMet 1.1 (http://bacmet.biomedicine.gu.se; Pal et al., 2014) and virulence genes were determined using the VFDB (http://www.mgc.ac.cn/VFs/; Chen et al., 2016) and BIGSdb (http://bigsdb.web.pasteur.fr/klebsiella/; Bialek-Davenet et al., 2014). Sequences from these databases were identified within the genomes of all isolates using BLAST+ (version 2.5.0; Morgulis et al., 2008) and Biopython (version 1.68; Cock et al., 2009). Multilocus sequence typing (MLST) was performed *in silico* using the *K. pneumoniae* database available from Pasteur Institute (http://www.pasteur.fr/mlst). K-loci were identified using Kaptive (Wyres et al., 2016).

Raw sequencing data has been deposited in the SRA under the BioProject PRJNA396729, Table S1 indicates the SRA number for each isolate.

Amino acid substitutions in the quinolone resistant-determining regions (QRDR), master regulators, RND efflux pump *acrAB-tolC*, outer membrane proteins, biofilm and cellulose genes (Bruchmann et al., 2015) were identified by comparing the genes against a representative of each ST type. Genomes were extracted from Pasteur database (http://bigsdb.pasteur.fr/klebsiella/klebsiella.html) and their ID references are as follows: ST340-ID1558; ST15-ID1698; ST14-ID1526; and ST147-ID3031.

## Results

### Bacterial isolates characterization

**Figure 1:**
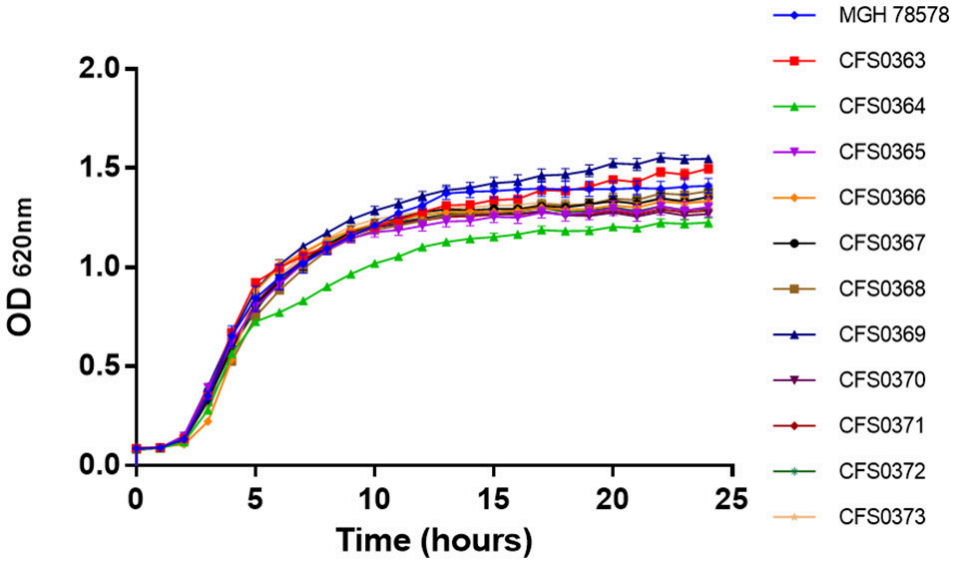
Growth curve traces for all 11 *Klebsiella pneumoniae* isolates in Mueller-Hinton broth at 37°C with shaking at 200 rpm.

A collection of 11 *K. pneumoniae*, of clinical origin were cultured in 2008, from a major tertiary teaching hospital in Dublin. These were initially characterized for their growth ability to grow in presence of a rich culture medium, Müeller-Hinton (MH) broth. Based on their growth curve profiles (Figure 1) all were capable of grow approximately at the same rate with generation times ranging between 49 and 51 min with isolate *K. pneumoniae* CFS0364 showing a less pronounced stationary phase. On occasion, clinical isolates may be misidentified when using traditional laboratory diagnostic methods. In order to confirm the bacterial genus, all of the 11 isolates were inoculated into GEN III MicroPlates™ and incubated in a Biolog platform (Biolog Inc., CA, USA), for identification. These data presented in Table 1 identified 5 of the 11 isolates (45% of the collection) as *K. pneumoniae*, the remaining isolates could not be reliably identified. The principles underpinning the Biolog identification protocol are based on the ability of bacterial isolates to metabolize a defined set of substrate compounds. Activation of certain metabolic pathways is related to the stress environment in which bacteria can be found. Therefore, clinical isolates may express a phenotype that does not necessarily lend itself to identification, under these conditions.

**Table 1:** A table showing clinical isolates identified using both GEN III ID plates and MALDI-TOF MS spectra (see also Figures S6.1–S6.11).

***Klebsiella pneumoniae***	**GEN III ID plates**	**MALDI-TOF ID**
CFS0363	*Klebsiella pneumoniae*	*Klebsiella pneumoniae*
CFS0364	*Klebsiella pneumoniae*	*Klebsiella pneumoniae*
CFS0365	*No ID*	*Klebsiella pneumoniae*
CFS0366	*Klebsiella pneumoniae*	*Klebsiella pneumoniae*
CFS0367	*No ID*	*Klebsiella pneumoniae*
CFS0368	*Klebsiella pneumoniae*	*Klebsiella pneumoniae*
CFS0369	No ID	*Klebsiella pneumoniae*
CFS0370	No ID	*Klebsiella pneumoniae*
CFS0371	No ID	*Klebsiella pneumoniae*
CFS0372	No ID	*Klebsiella pneumoniae*
CFS0373	*Klebsiella pneumoniae*	*Klebsiella pneumoniae*

*The isolates unable to be identified by metabolic profiles were highlighted in yellow*.

To further resolve this identification, all isolates were subjected to further analysis by MALDI-TOF mass spectrometry using the VITEK® MS platform. This strategy is faster and more accurate when compared with Biolog, results obtained are presented as in Table 1. These data indicated that all these clinical isolates were identified as *K. pneumoniae* with 99.9% confidence.

### Antimicrobial susceptibility testing (AST)

**Figure 2:**
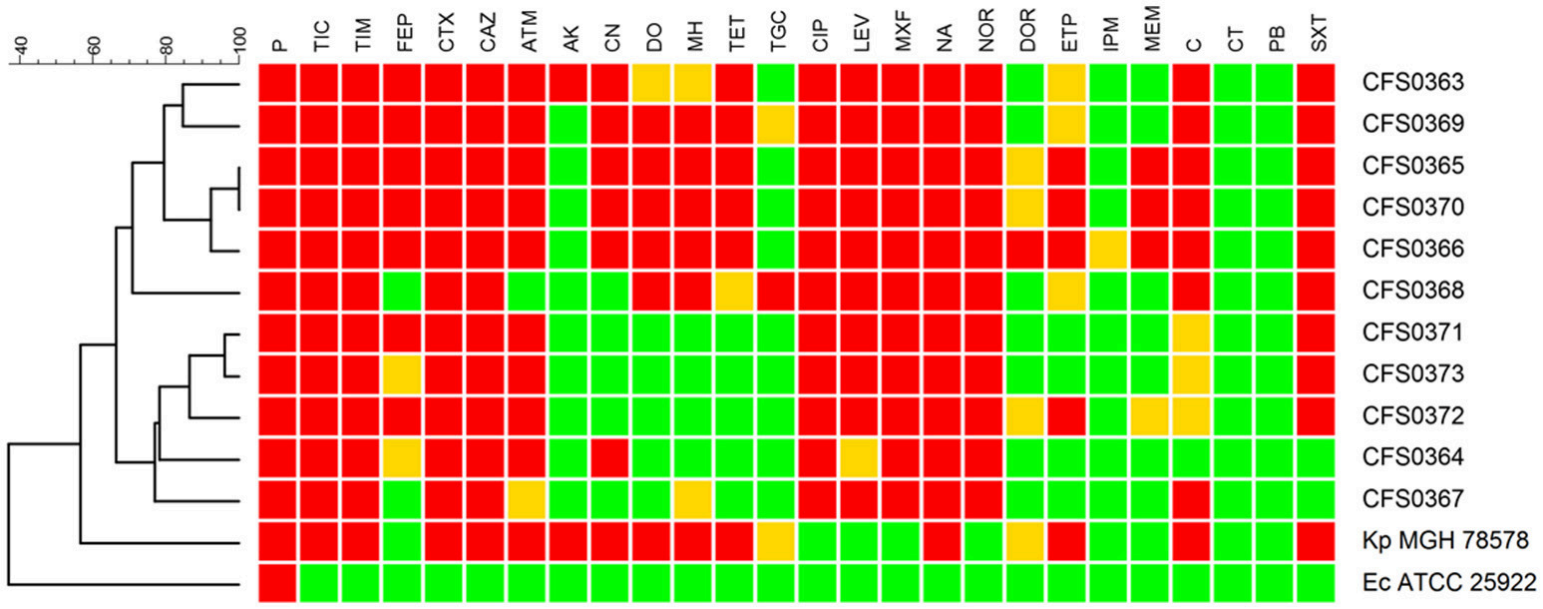
A dendrogram, constructed using the BioNumerics™ software (ver 7.5, Applied Maths), showing the antibiotic resistance profiles obtained following disk diffusion assays. The dendrogram shows two main clusters based on these resistance profiles. Breakpoints were interpreted according to CLSI guidelines 2013 with the following exceptions: Tigecycline, Moxifloxacin—the breakpoint used was that shown in the EUCAST guidelines 2016. Colistin and Polymyxin B—breakpoints were determined using the broth microdilution method and interpreted using EUCAST guidelines 2016. The following abbreviations apply to the listing compounds within drug classes: P, Penicillin; TIC, Ticarcillin; TIM, Ticarcillin-clavulanic acid; FEP, Cefepime; CTX, Cefotaxime; CAZ, Ceftazidime; ATM, Aztreonam; CN, Gentamicin; AK, Amikacin; DO, Doxycycline; MH, Minocycline; TE, Tetracycline; TGC, Tigecycline; CIP, Ciprofloxacin; LEV, Levofloxacin; MXF, Moxifloxacin; NA, Nalidixic Acid; NOR, Norfloxacin; DOR, Doripenem; ETP, Ertapenem; IPM, Imipenem; MEM, Meropenem; C, Chloramphenicol; CT, Colistin sulfate; PB, Polymyxin B; SXT, Trimethoprim-Sulfametoxazole.

To extend the characterization of these *K. pneumoniae* their susceptibility to a panel of antimicrobial compounds was studied. These assays were performed using both disk diffusion including a set of 26 antimicrobial compounds containing representatives of each class and broth microdilution. Based on the resistance profiles obtained following disk diffusion, the study collection of *K. pneumoniae* demonstrated two distinct clusters (Figure 2). All the isolates were found to be resistance to β-lactam-based compounds including, cephalosporins, along with quinolones and fluoroquinolones. Resistance to chloramphenicol accounted for 64% (*n* = 7) of the collection and trimethoprim-sulfametoxazole resistance was detected in 9 of 11 isolates. One of the two clusters identified (including the isolates *K. pneumoniae* CFS0363, CFS0369, CFS0365, CFS0370, CFS0366, and CFS0368) was found to be resistance to tetracyclines, aminoglycosides (with exception of the isolate *K. pneumoniae* CFS0368), and some of the carbapenem compounds tested. The second cluster, comprising of the remaining isolates, was mainly characterized by its susceptibility to aminoglycosides with the exception of *K. pneumoniae* CFS0364 that was found to be resistant to gentamicin. In contrast to the above, the latter cluster was also notable by its susceptibility to tetracyclines.

In the case of the three carbapenem compounds tested, all isolates were susceptible, with the exception of *K. pneumoniae* CFS0372 which was found to be resistant to ertapenem and also demonstrated intermediate resistance to meropenem and doripenem.

To further validate the disk diffusion data, broth microdilution was also performed. These data are shown in Table 2. Resistance to ampicillin was a characteristic feature of Enterobacteriaceae and was used as an internal control. All clinical isolates were found to be resistant to cephalosporins, quinolones, and fluoroquinolones whereas only 54 and 45% of the isolates expressed resistance to gentamicin and tetracycline, respectively. The isolates were also tested against a benzalkonium chloride, a common quaternary ammonium compound used as a disinfectant. Results (Table 2) show that the MICs varied from 8 to 32 μg/mL in accordance with previous reports (Abuzaid et al., 2012; Guo et al., 2015).

**Table 2:** Minimal Inhibitory concentrations (MIC) and minimal bactericidal concentrations (MBC) obtained for *Klebsiella pneumoniae* clinical study isolates, tested against a panel of 11 antimicrobial compounds.

***Klebsiella pneumoniae***	**Antimicrobial susceptibility—Minimal inhibitory concentration (MIC) and minimal bactericidal concentration (MBC) (**μ**g/mL)**																						**No. of resistant drug classes**
	**Ampicillin**		**Cefotaxime**		**Gentamicin**		**Kanamycin**		**Tetracycline**		**Imipenem**		**Nalidixic acid**		**Ciprofloxacin**		**Moxifloxacin**		**Chloramphenicol**		**Benzalkonium chloride**		
	**MIC**	**MBC**	**MIC**	**MBC**	**MIC**	**MBC**	**MIC**	**MBC**	**MIC**	**MBC**	**MIC**	**MBC**	**MIC**	**MBC**	**MIC**	**MBC**	**MIC**	**MBC**	**MIC**	**MBC**	**MIC**	**MBC**	
MGH 78578	>512	>512	16	16	64	128	>512	>512	>512	>512	1	1	>512	>512	16	16	1	1	512	>512	16	16	6
CFS0363	>512	>512	256	256	128	256	256	256	128	256	1	1	>512	>512	64	64	16	16	>512	>512	8	8	6
CFS0364	>512	>512	128	128	128	128	4	8	1	2	1	1	>512	>512	8	8	4	4	2	4	16	16	4
CFS0365	>512	>512	>512	>512	128	128	8	8	>512	>512	1	1	>512	>512	32	32	16	16	>512	>512	16	16	6
CFS0366	>512	>512	>512	>512	256	256	16	16	>512	>512	2	2	>512	>512	32	32	32	16	>512	>512	16	16	6
CFS0367	512	512	16	16	1	1	1	1	2	4	1	2	>512	>512	8	8	8	8	32	32	16	16	4
CFS0368	512	512	8	8	1	1	1	1	8	8	1	1	>512	>512	8	8	8	8	256	256	32	32	4
CFS0369	>512	>512	>512	>512	64	64	8	8	512	512	1	1	>512	>512	16	16	16	16	>512	>512	16	16	6
CFS0370	>512	>512	>512	>512	128	128	8	8	>512	>512	1	2	>512	>512	32	32	32	32	>512	>512	16	16	6
CFS0371	>512	>512	128	128	1	1	1	1	1	2	1	1	>512	>512	16	16	16	16	32	64	8	8	4
CFS0372	>512	>512	256	256	1	1	2	2	2	2	1	1	>512	>512	32	32	32	32	32	32	16	16	4
CFS0373	>512	>512	128	128	1	1	2	2	1	1	1	1	>512	>512	16	16	16	16	64	64	8	8	4

*Highlighted in yellow is the number of resistant antimicrobial classes for each isolate. An isolate resistant to more than 3 different classes was considered multi-drug resistant (MDR)*.

Results obtained using both of these AST-based protocols, indicate that all eleven bacterial isolates can be defined as multi-drug resistant (MDR), since they were simultaneously resistant to three or more classes of antimicrobial compound. Study collection isolates were found to be resistant to 4 or more and up to 8 classes of dug.

### Assessment of membrane-mediated efflux

Efflux ability contributes for the increased bacterial resistant phenotype. Isolates were compared for their ability to extrude ethidium bromide (EtdBr) from the cell, revealing thus their efflux potential. In this method cells were saturated with EtdBr in a saline solution using CCCP to facilitate the uptake of EtdBr at a concentration that did not affect the cell viability (Srinivasan and Rajamohan, 2013).

**Figure 3:**
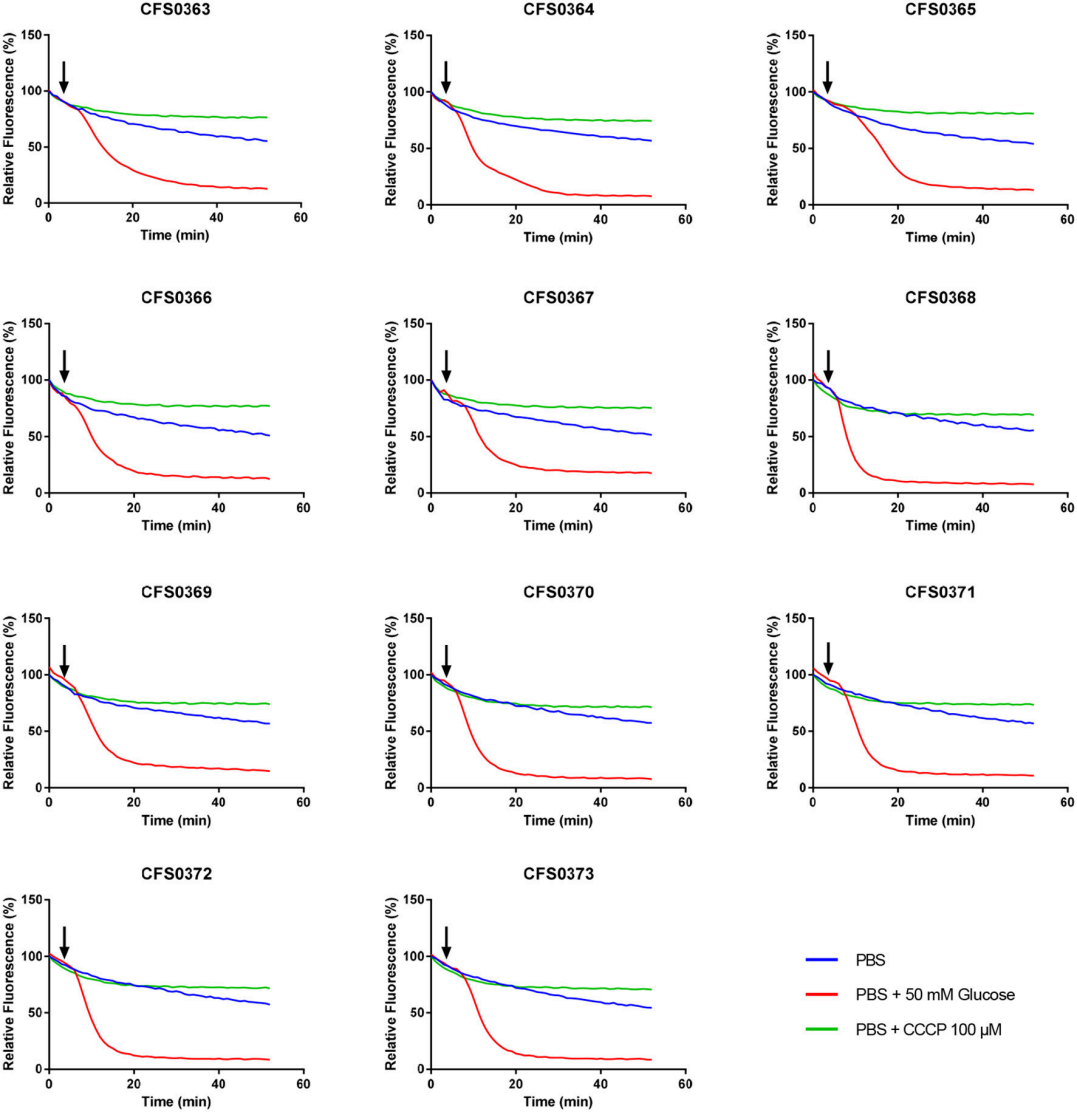
Determination of the ethidium bromide efflux by a semi-automated fluorometric method. *Klebsiella pneumoniae* isolates were saturated with 50 μM ethidium bromide and efflux measured by fluorometry at 37°C for 50 min with an excitation and emission wavelengths of 518- and 606-nm, in the presence or absence of glucose and CCCP. Black arrows indicate when glucose (50 mM) was added after 3 min of initial reading.

The capacity for cells to extrude EtdBr is improved in the presence of glucose, as the latter energizes the bacterial cell. The over-expression of efflux pumps is indicated by a fast decrease in fluorescence in both the presence or absence of glucose. The results obtained (Figure 3) indicates no clear differences in the efflux activity of these clinical isolates when compared among themselves.

### Biofilm formation

The ability to form biofilms plays a critical role in a bacterium's ability to colonize the host or any indwelling medical devices temporarily, *in situ*. This phenotype was studied by measuring bacterial growth in the presence of a rich culture media (in this case MH broth; see Figures S1, S2) and in a media designed to stress these isolates, M9 minimal media (see Figures S3, S4). Isolates when inoculated into these culture media, were maintained statically and incubated at 25 and at 37°C for 96 h.

Biofilm formation in MH decreased over time at 25°C, whereas at 37°C it appeared to decrease for the first 48 h and then increased, back to the initial values recorded until 96 h during static growth. In contrast, biofilm formation in M9 minimal media increased over time at both 25 and 37°C, indicating a better adaptation when subjected to a laboratory induced stress condition. The effect of temperature on biofilm formation in MH was stronger at 25°C during the first 48 h, for all isolates with the exception of *K. pneumoniae* CFS0364 (this isolate was comparatively weaker both at 25 and 37°C). Biofilm maturation was observed to be better in MH at 37°C after 48 h. In M9 minimal media biofilm formation was stronger at 37°C with exception of the isolate *K. pneumoniae* CFS0363 that exhibited better growth at 25°C. Overall, with the exception of *K. pneumoniae* CFS0372, the remaining isolates produced more biofilm when cultured in M9 minimal media than when grown on MH at 37°C. Moreover, the effect of stress conditions appeared to promote biofilm formation in the case of isolate *K. pneumoniae* CFS0364 at 37°C, indicating a potential adaptive survival response.

The expression of curli fimbriae, by bacteria is known to be involved in adhesion of these cells to surfaces, and it is also associated with cell aggregation and biofilm development. Cellulose is an extracellular matrix component of biofilms formed by bacteria of the Enterobacteriaceae family (Zogaj et al., 2001). When cellulose is produced together with these curli fimbriae, both produce a highly inert, hydrophobic extracellular matrix around the bacterium enhancing colonization capacity (Bokranz et al., 2005). The production of these curli fimbriae was determined by observing colony morphology on Congo red and calcofluor agar plates. Morphotypes were categorized according to Zogaj et al. (2001). The growth of a *red, dry, and rough (RDAR)* colony on Congo red agar plates indicated the presence of curli fimbriae whilst fluorescence detection in the calcofluor plates under UV light indicated cellulose production. Disruption or down-regulation of the corresponding genes, would provide bacteria with a distinct *smooth and white (SAW)* morphotype. A deficiency in curli fimbrial expression would render the colonies *pink dry and rough (PDAR)*.

The results shown in Tables 3 and 4 provide images of the morphotypes obtained when isolates were grown in MH agar with the corresponding dyes at 37 and 25°C, respectively, as well as the pellicle formation, that occurred under the same conditions. Interestingly, results at 37°C did not show any distinctive morphotypes that would suggest the expression of curli fimbriae. The majority of the isolates showed a smooth-textured red-like morphotype (RAS—red and smooth). The same observations were also reported in *Klebsiella* species isolated from human gastrointestinal tract (Zogaj et al., 2003).

**Table 3:** Congo red and calcofluor morphotypes obtained for *Klebsiella pneumoniae* grown in Müeller-Hinton agar at 37°C for 72 h.

***Klebsiella pneumoniae***	**ST 14028**	**MGH 78578**	**CFS0363**	**CFS0364**	**CFS0365**	**CFS0366**	**CFS0367**	**CFS0368**	**CFS0369**	**CFS0370**	**CFS0371**	**CFS0372**	**CFS0373**
Congo Red 37°C	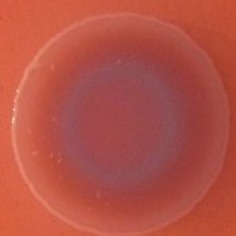	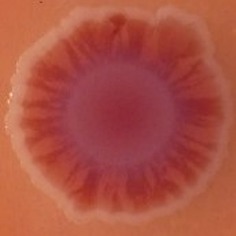	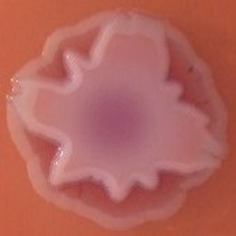	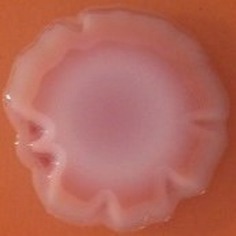	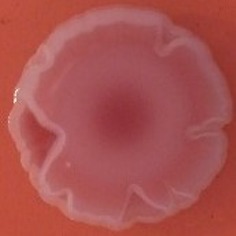	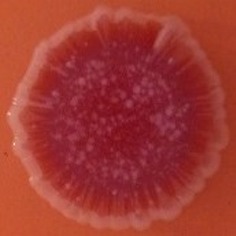	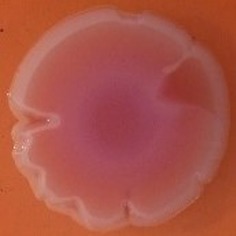	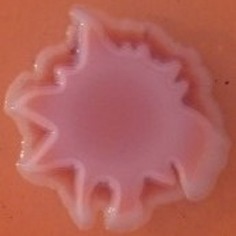	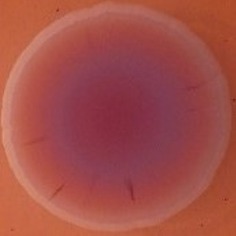	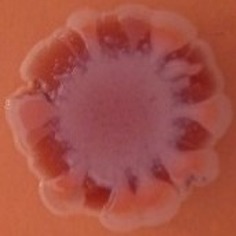	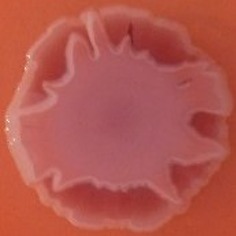	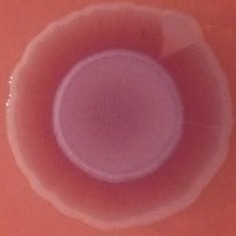	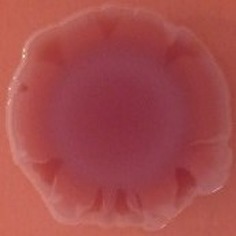
Calcofluor 37°C	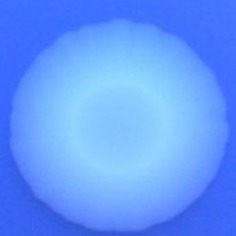	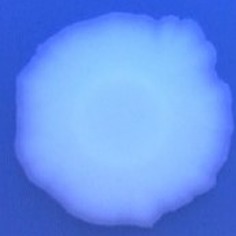	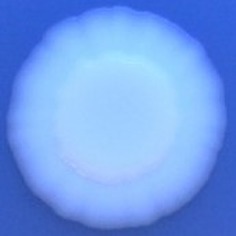	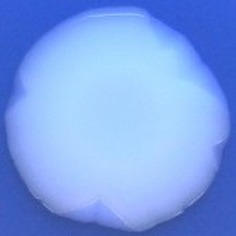	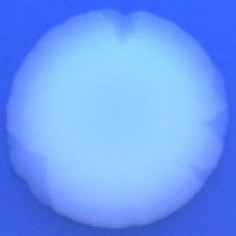	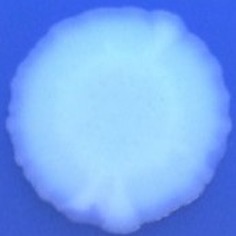	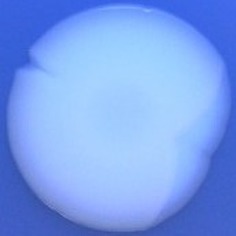	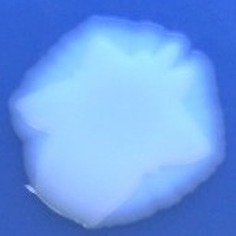	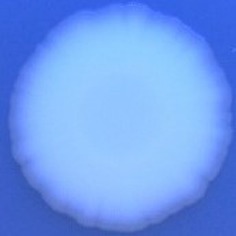	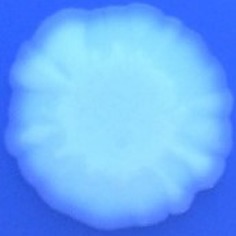	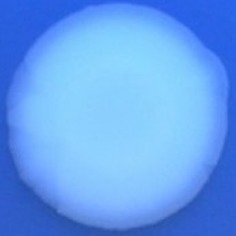	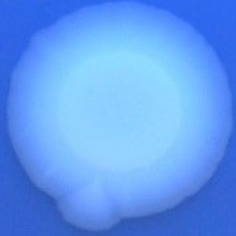	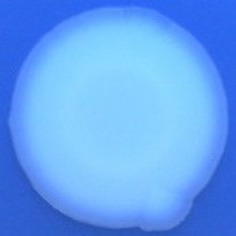
Pellicle 37°C	N/A	+	+	+	−	−	+	+	+	+	+	+	+
Morphotype	SAW	RAS	SAW	SAW	SAW	RAS	SAW	SAW	SAW	SAW	SAW	SAW	SAW
Clonal group	N/A	CG60	CG147	CG15	CG258	CG258	CG15	CG15	CG258	CG258	CG258	CG258	CG258

*Pellicle formation at the liquid-air interface is indicated as “+” presence (and highlighted in yellow) and “−”absence. Morphotypes: SAW, smooth and white; RDAR, red dry and rough; RAS, red and smooth*.

**Table 4:** Congo red and calcofluor morphotypes obtained for *Klebsiella pneumoniae* grown in Müeller-Hinton agar at 25°C for 72 h.

***Klebsiella pneumoniae***	**ST 14028**	**MGH 78578**	**CFS0363**	**CFS0364**	**CFS0365**	**CFS0366**	**CFS0367**	**CFS0368**	**CFS0369**	**CFS0370**	**CFS0371**	**CFS0372**	**CFS0373**
Congo Red 25°C	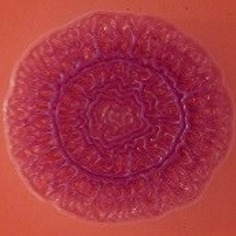	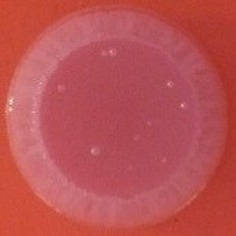	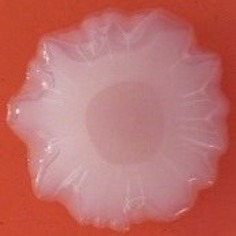	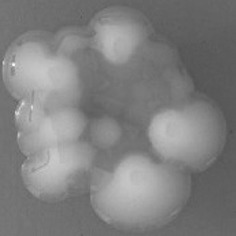	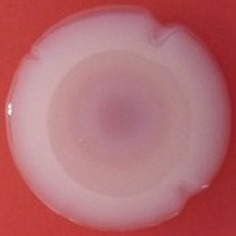	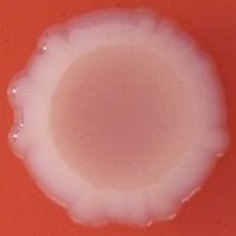	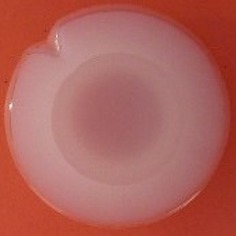	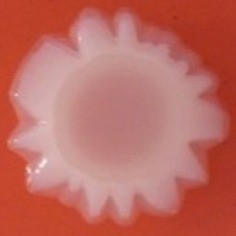	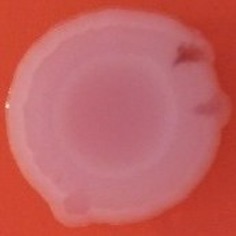	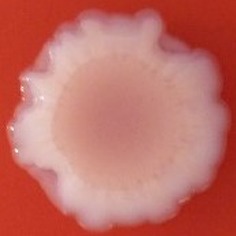	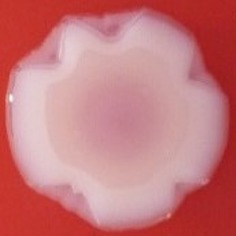	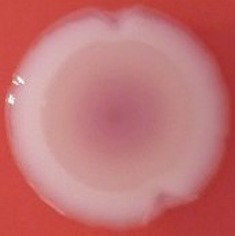	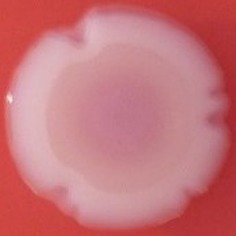
Calcofluor 25°C	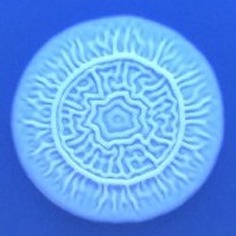	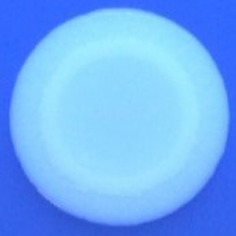	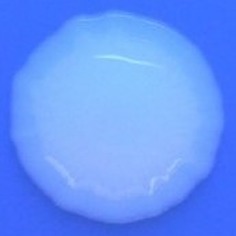	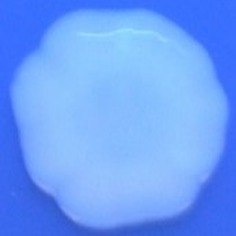	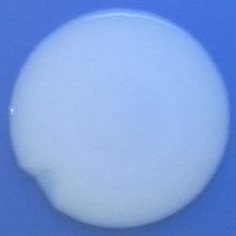	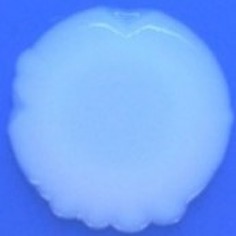	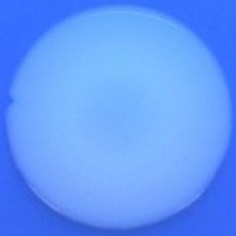	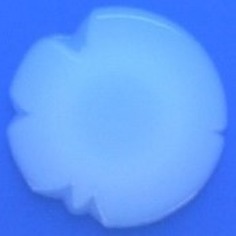	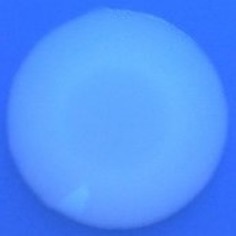	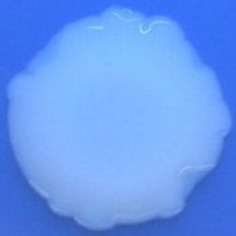	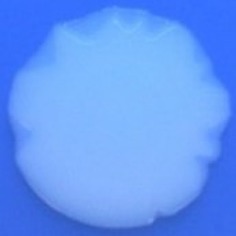	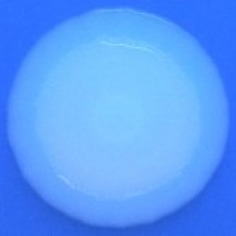	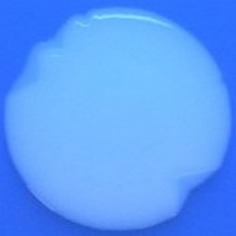
Pellicle 25°C	N/A	+	+	+	−	−	+	+	−	−	−	−	−
Morphotype	RDAR	SAW	SAW	SAW	SAW	SAW	SAW	SAW	SAW	SAW	SAW	SAW	SAW
Clonal group	N/A	CG60	CG147	CG15	CG258	CG258	CG15	CG15	CG258	CG258	CG258	CG258	CG258

*Pellicle formation at the liquid-air interface is indicated as “+” presence (and highlighted in yellow) and “−” absence. Morphotypes: SAW, smooth and white; RDAR, red dry and rough*.

Production of cellulose was studied when isolates were cultured at 37°C and in this case *K. pneumoniae* CFS0366, CFS0368, CFS0370, and CFS0373 exhibited a notable expression of cellulose. The formation of cell aggregates at the air-liquid interface, was also determined in MH broth. With exception of *K. pneumoniae* CFS0365 and CFS0366 all were found to form a pellicle at 37°C.

When isolates were grown at 25°C they all exhibited a similar SAW morphotype indicating the absence of curli fimbriae and production of cellulose. The formation of a pellicle at 25°C was only associated with the isolates *K. pneumoniae* CFS0363, CFS0364, CFS0367, and CFS0368, all of which belonged to the CG147 and CG15.

In general, the morphotypes observed at 25°C were broadly consistent with the data obtained for biofilm formation. Data obtained when these isolates were cultured at 37°C was inconclusive and it was not possible to correlate, these findings with the biofilm formation.

When the genome sequences were queried, amino acid substitutions were identified in several genes involved in biofilm formation in *K. pneumoniae* CFS0367 and CFS0368 of the CG15 (see Table S3). The genes included *astC*, a succinylornithine transaminase, *astD* a succinylglutamic semialdehyde dehydrogenase, both involved in proline and arginine metabolism. *ulaA* (a component of the ascorbate-specific PTS system, EIIC), *yfiB* that codes for an internal membrane protein and *yfiN*, a diguanylate cyclase, involved in the regulation of type-3 fimbriae and cellulose production. These two isolates show differences when the biofilm formation is compared in the different *in vitro* conditions (with the exception of Müeller-Hinton at 25°C), possibly due to the substitution found on *yfiN* of *K. pneumoniae* CFS0366 that show impaired ability to form biofilm.

### Bacterial whole genome sequence (WGS) determination and downstream bioinformatic analysis

All 11 *K. pneumoniae* were sequenced using the Illumina HiSeq platform and found to have an average genome size of ~5.4-Mbp with the exception of *K. pneumoniae* CFS0363 that produced a larger genome size of 5.8-Mbp (Table S1 and Figure S5). For the majority of the isolates, an average of 5,000 coding DNA sequences (CDS) were identified, and in the case of the isolate *K. pneumoniae* CFS0363 this number was higher as a consequence of the larger genome size. An overview of the assembly metrics is presented in Table S1.

**Figure 4:**
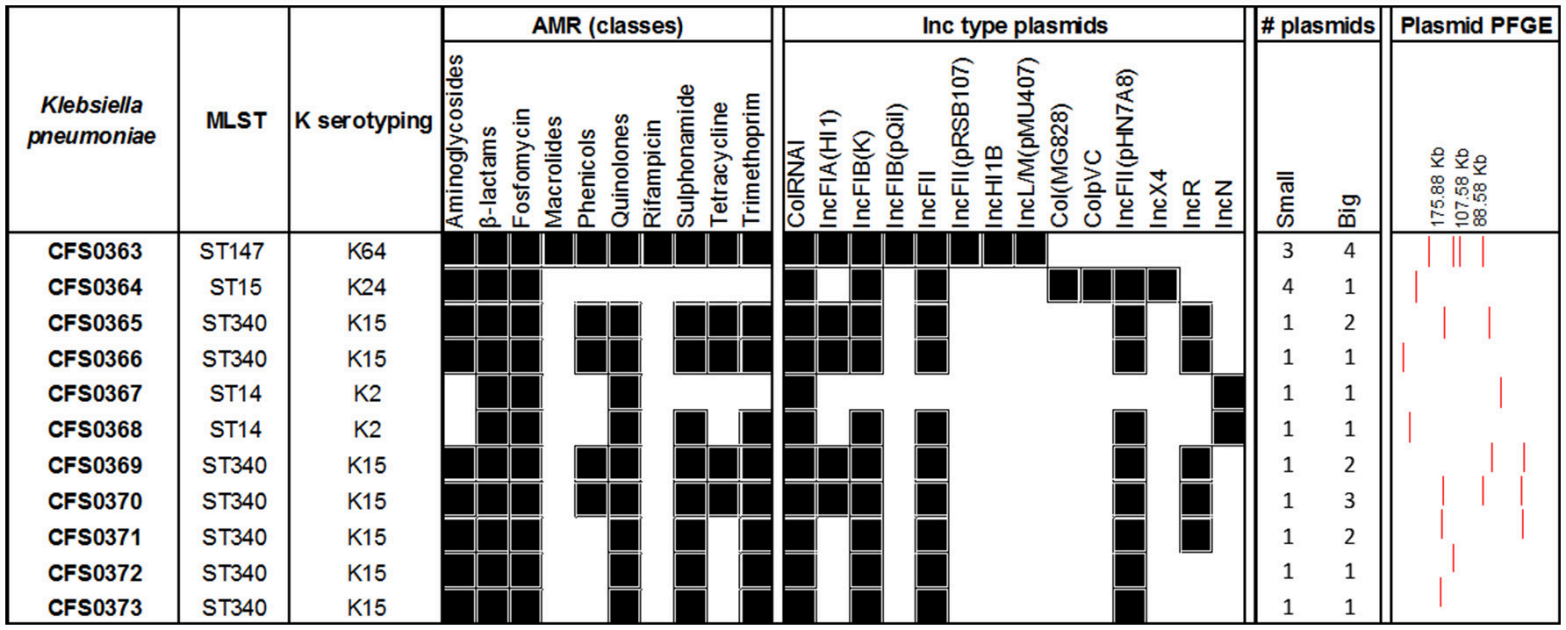
Genomic analysis of *Klebsiella pneumoniae* clinical isolates. Antimicrobial resistance genes were grouped by class and the presence of at least one gene was considered as a positive result (black). The number of plasmids was identified from the gels (large plasmids >25 kb, small plasmids 2–25 kb).

*In silico* MLST was performed for all the isolates (Figure 4) using the seven-marker gene set (*gapA, infB, mdh, pgi, phoE, rpoB, tonB*), and sequence types (STs) were assigned using the Institute Pasteur scheme. The majority of the isolates (64%) belonged to ST340 (serotype K15) which is part of the widely disseminated clonal group CG258, associated with ESBL- and carbapenem-resistance (Chen et al., 2014). Three of the eleven isolates (*K. pneumoniae* CFS0364, CFS0367 and CFS0368) belonged to the epidemic clonal group CG15. Further, *K. pneumoniae* CFS0364 (ST15) had the capsular serotype K24 whilst *K. pneumoniae* CFS0367 and CFS0368 (ST14) were associated with the K2 capsular serotype. The isolate *K. pneumoniae* CFS0363 presented the capsular serotype K64 and the sequence type ST147 which belongs the CG147 cluster, and is considered to be an epidemic resistant clonal group (Damjanova et al., 2008; Baraniak et al., 2013).

The presence of acquired antimicrobial resistance-encoding genes was determined using the resistance database Resfinder. The majority of the isolates (82%) harbored the ESBL-encoding gene *bla*_CTX-*M*-15_ (Table S2). The genes *bla*_SHV-12_ and *bla*_TEM-1*B*_ was also identified in 56 and 45% of the isolates, respectively. A resistance gene for fosfomycin (*fosA*) was present in all the isolates and the efflux pump *oqxAB*, responsible for fluoroquinolone resistance, was identified in 10 of the 11 isolates (being absent in isolate *K. pneumoniae* CFS0364). Moreover, resistance genes to phenicols (*catA* and *catB*) and tetracyclines (*tet*) accounted for 45% of the isolates in both cases. Sulfonamide and trimethoprim resistance-encoding genes were identified in 82% of the isolates.

Interestingly, the isolates from the epidemic group CG15 showed a more susceptible phenotype when compared with the remaining isolates of the collection (Figure 2). A common resistance determinant that was identified in all the isolates of this clonal group was the ESBL resistance gene *bla*_SHV-28_. Additionally, isolates *K. pneumoniae* CFS0367 and CFS0368 also harbored the AmpC β-lactamase gene *bla*_ACC-1_, which has been reported to be associated with nosocomial outbreaks in Europe (Ohana et al., 2005; Roche et al., 2008). The isolate *K. pneumoniae* CFS0363 possessed a number of β-lactam-related resistance encoding genes including *bla*_CMY-4_, *bla*_OXA-1_, *bla*_OXA-9_, *bla*_SHV-12_, and *bla*_TEM-1*A*_. No genes were identified conferring resistance to carbapenems.

The presence of the erythromycin resistance genes *ere(A)* and *ere(B)* and the plasmid-mediated quinolone resistance (PMQR) gene *qnrB1* was also confirmed in the isolate *K. pneumoniae* CFS0363. Resistance to quinolones and fluoroquinolones can also be due to target gene mutations in *gyrA, gyrB, parC*, and *parE* (Casin et al., 2003; Baucheron et al., 2004; Fu et al., 2013). Table S3 presents a summary of the mutations identified in these proteins and those involved in efflux and regulation. *K. pneumoniae* CFS0367 and CFS0368 have the same amino acid substitutions in GyrA that were identified at position 83 (F83Y), position 87 (G87D) and a deletion found at position 48. Similarly, *K. pneumoniae* CFS0364 contained a GyrA amino acid substitution consistent with the positions 645 (N645H) in the polypeptide chain. The *K. pneumoniae* isolate CFS0367 was found to have one amino acid substitutions in each of the remaining proteins GyrB, ParC, and ParE, at positions 466 (E466D), 84 (G84E), and 460 (Q460P) respectively. In contrast, only one amino acid substitution in the protein sequence of ParC was found in *K. pneumoniae* CFS0368 at position 84 (G84E). All remaining isolates of the clonal group CG147 and CG258 were devoid of mutations when compared with the respective reference of their ST type. These mutations are often accompanied by the acquisition of additional resistance-encoding genes identified along with the overexpression of efflux pumps *oqxAB* and *kexD* known to contribute to fluoroquinolone resistance. KexD efflux pump was identified in 73% (*n* = 8) of the isolates studied (see Table S2). Remarkably, this pump was absent in those isolates from the clonal group CG15. The efflux pump *oqxAB* was absent in the isolate *K. pneumoniae* CFS0364.

Mutations in efflux systems and global regulators could also contribute to the resistance phenotype. Amino acid sequence analysis of the RND efflux pump AcrAB-TolC identified a truncation in the regulator AcrR for the isolate *K. pneumoniae* CFS0367 and an amino acid substitution of at the position 715 (R715L) for the isolate *K. pneumoniae* CFS0368. Moreover, one residue was found to be deleted in the regulator RamR in *K. pneumoniae* CFS0363 and CFS0368. Interestingly, in the isolates *K. pneumoniae* CFS0364 and CFS0367 the *ramRA* regulon were not detected.

To gain an insight into the nature of any plasmids, contained in these isolates, replicon typing was performed *in silico* using PlasmidFinder (Figure 4). All were positive for the colRNAI type, a common replicon associated with small plasmids found in *Klebsiella* species (Ramirez et al., 2014). The virulence-encoded incompatibility types IncFIB(K) and IncFII were identified in 91% of the isolates whereas IncR and IncFIA accounted for 45% of these isolates. IncF plasmids are often associated with ESBL-resistance encoding genes (Carattoli, 2009; Doumith et al., 2012; Markovska et al., 2014). Moreover, the isolate *K. pneumoniae* CFS0363 an isolate with four large plasmids also harbored Inc types including IncFIB(pQil), IncHI1B and the IncL/M. These replicon types are widely disseminated in the environment and often associated with MDR non-typhoidal and typhoidal *Salmonella* species and with carbapenem resistance, respectively (Holt et al., 2011; Zurfluh et al., 2014; Kubasova et al., 2016; Ouertani et al., 2016; Pérez-Vázquez et al., 2016).

The isolate *K. pneumoniae* CFS0364 was the only bacterium harboring the colpVC and IncX4 replicon types which are known to be associated with *bla*_CTX-*M*_-mediated resistance both in humans and animals (Johnson et al., 2012; Lo et al., 2014). Recently, these plasmids were also epidemiologically linked with the carriage of the colistin resistance gene *mcr-1* (Fernandes et al., 2016). Colistin resistance was not detected among any of the isolates studied. Interestingly, the IncN replicon type was only associated with the K2 serotype isolates *K. pneumoniae* CFS0367 and CFS0368. The latter replicon type was also associated with the ESLB resistance gene *bla*_SHV-12_ (Markovska et al., 2014) and carbapenamase resistance (Rodrigues et al., 2016; Tijet et al., 2016).

The presence of virulence genes may influence the capacity that these pathogens have on effective host colonization and infection. Thus, it is relevant to gain some understanding of this potential through the identification of such genes, in particular the iron scavenging genes that are known to be associated with invasive disease (Holt et al., 2015). When analyzed, all isolates in this study (Table S2) harbored the enterobactin iron uptake system (*ent* and *fep* operon), a ubiquitous system found in *K. pneumoniae* (Paczosa and Mecsas, 2016). Isolates *K. pneumoniae* CFS0363 and CFS0364 also presented the yersiniabactin siderophore system (*fyuA, irp1, irp2* and *ybt* operon). The isolates of the clonal group CG15 all presented the *kfuabc* operon. These iron-scavenging operons have been associated with invasive *K. pneumoniae* isolates (Ma et al., 2005; Bachman et al., 2012; Holt et al., 2015). One peculiarity of the isolate *K. pneumoniae* CFS0363 was the presence of the metal-resistance tellurium operon that was identified, in contrast to its complete absence in the remaining isolates.

All the isolates possessed several biocide resistance-encoding genes that were detected *in silico* using BacMet. The quaternary ammonium resistance-encoding gene *qacE*Δ*1*, commonly associated with class 1 integrons, was identified in all isolates but was found to be absent in the *K. pneumoniae* CFS0364 and CFS0367. The remaining biocide resistance genes is summarized in Table S2.

## Discussion

*K. pneumoniae* has been described over the past decades as an opportunistic pathogen associated with the healthcare setting wherein it is capable of causing severe illnesses. The continuous dissemination of these pathogens was for many years disregarded until now. As a consequence, *Klebsiella* is now considered a reservoir for antimicrobial resistance and virulence genes (Holt et al., 2015). The acquisition and dissemination of ESBL-, carbapenem- and now colistin-resistance encoding genes (Gu et al., 2016; Ridolfo et al., 2016) makes this pathogen an urgent threat to human health. In this study, a collection of *K. pneumoniae* isolates resistant to ESBL- and FQ- compounds were further characterized.

All the isolates analyzed in this study were members of one or other of the epidemic and international MDR clonal groups CG258, CG15, or CG147. The majority of the sequence types identified (ST340) belonged to the CG258. This sequence type has been mainly associated with the presence of genes *bla*_KPC_, *bla*_NDM_ conferring resistance to carbapenems in different countries including Spain (Sánchez-Romero et al., 2012; Pérez-Moreno et al., 2016), Greece (Giakkoupi et al., 2011), Russia (Ageevets et al., 2014), the UK and Sweden (Giske et al., 2012), South Korea (Kim et al., 2012), South America (Martins et al., 2015; Cerdeira et al., 2016a,b; Horna et al., 2017), and others. Isolates of the ST340 in this collection do not possess any recognized acquired carbapenem resistance-encoding gene, despite the fact that, three of them (*K. pneumoniae* CFS365, 366-, and 370) were phenotypically resistant to meropenem and ertapenem. The remaining ST types identified belonged to the epidemic CG15 and CG147 that have been identified worldwide (Lee et al., 2016). The CG15 is known for harboring the hypervirulent strains capable of causing serious illness. Moreover, two isolates (of sequence types ST147 and ST15) contained the yersiniabactin siderophore-encoding genes a characteristic iron-scavenging mechanism of invasive *K. pneumoniae* (Holt et al., 2015).

Resistance genes related to ESBL-type drugs were identified as *bla*_CTX-*M*-15_, *bla*_SHV-12_, and *bla*_TEM-1*B*_ representing 82, 55, and 45% of the collection, respectively. These resistance-encoding genes are found widely spread in the international context, in particular among the members of the Enterobacteriaceae family (Tal Jasper et al., 2015; Arcilla et al., 2017). Resistance-encoding genes to other classes of antimicrobial compound including aminoglycosides, fosfomycins, macrolides, tetracyclines, sulfonamides, and trimethoprim were also detected.

Resistance to fluoroquinolones was found to be associated with several mutations mapped to the QRDR or efflux pump-encoding genes. The presence of QRDR mutations was found for all the isolates when compared with the reference isolate *K. pneumoniae* MGH 78578 (data not shown). In contrast, however when QRDR genes were compared against isolates of the same ST type most of these isolates appeared to possess these same polymorphisms. In contrast, mutations were identified in isolates of the CG15 group (Table S3). These data suggested that specific QRDR mutations may be more common among particular ST types.

Resistance to quinolones and fluoroquinolones can also be caused by several other mechanisms including efflux pump systems, such as *oqxAB* which is known to be associated with low-level fluoroquinolone resistance (Rodriguez-Martinez et al., 2013). This mechanism was identified in almost all isolates with the exception of *K. pneumoniae* CFS0364 (ST15). Other efflux mechanisms identified included the pump *kexD*, which interestingly was absent only in isolates of the clonal group CG15. MIC values for ciprofloxacin and moxifloxacin measured for isolates of the CG15 were lower compared to other isolates in this study collection. All the isolates were still considered resistant to fluoroquinolones, although the MIC values were not particularly high. A feature that might suggest moderate selection of resistance against these class of antimicrobial compounds.

Intrinsic efflux-resistance mechanisms can also contribute to those phenotypes observed. Interestingly, the ethidium bromide efflux patterns observed among the *K. pneumoniae* study collection did not show any differences despite the presence of various mutations in global regulatory genes, as well as in genes coding for efflux regulators and pumps found in *K. pneumoniae* CG15 and CG147 (Table S3). This feature would suggest the involvement of other redundant efflux-resistance mechanisms, that could play a role in the extrusion of EtdBr and antimicrobial compounds. Other efflux pumps including the RND efflux pump AcrD or MdtABC have also been reported to be involved in antimicrobial resistance in other pathogens of the Enterobacteriaceae family (Nishino et al., 2007). Moreover, the recent identification of KpnEF an efflux pump linked with resistance to several classes of antibiotics, dyes and detergents (Srinivasan and Rajamohan, 2013) or the efflux pump KmrA responsible for extrusion of several compounds such as kanamycin, norfloxacin, acriflavine, ethidium bromide, methyl viologen, tetraphenylphosphonium chloride, and others (Ogawa et al., 2006) could be responsible for the phenotype observed when using ethidium bromide as a substrate molecule.

Mutations identified in the outer membrane protein K36 (*ompK36*) in *K. pneumoniae* CFS0367 and CFS0368 (Table S3) could also influence the resistance of these isolates. This feature was not explored further.

The presence of broad host range plasmids was confirmed *in silico* following the identification of replicon types including IncFII, IncFIB, IncFIA, and IncR all common among the isolates. In addition, other replicon types such as IncL/M, IncX4, or IncN were associated with the *K. pneumoniae* isolates of CG147 and CG15 alone, but based on profiling no common plasmid was identified among these isolates. Similar results for these ST types were also reported previously (Damjanova et al., 2008; Rodrigues et al., 2014; Mansour et al., 2015). No clear relationship between replicon- and sequence types was observed among the remaining isolates in the collection.

The *qacE*Δ*1* gene, which functions to express resistance to quaternary ammonium compounds, including benzalkonium chloride was identified *in silico* (Paulsen et al., 1993). Susceptibility to the latter was measured for all of the 11 clinical isolates and MIC values obtained were found to be similar to those previously published (Abuzaid et al., 2012; Guo et al., 2015). The former pump along with other types of efflux systems plays an important role in enabling *K. pneumoniae* to adapt to various environmental stresses. Understanding the regulation of these mechanisms could further elucidate their role(s) in antimicrobial resistance.

Most of the study isolates exhibited the ability to form biofilms under stress conditions, as exemplified by the use of M9 minimal media at 37°C. In this case conditions isolates could increase their biomass over a period of 96 h a feature that could facilitate their persistence in the environment. Four isolates (*K. pneumoniae* CFS0363, CF0364, CFS0367, and CFS0372) belonging to different ST types, expressed a diverse ability to form a biofilm biomass. No underlying genotype could be identified that could explain the phenotype observed.

In conclusion, these data confirmed the fact that well-recognized clonal groups of *K. pneumoniae* of importance to human health carry a diverse repertoire of acquired and intrinsic antimicrobial resistance-encoding mechanisms. Some of these are related to resistance to compounds within critically important drug classes such as those of the ESBL and FQ group. When subjected to laboratory-induced stress, most of the study isolates formed strong biofilms, a phenotype that would be of importance to nosocomial infections deriving from medical devices, such as indwelling catheters. These findings once again confirm the challenge faced by public health professionals in overcoming this pathogen of importance to human health.

## Author contributions

Conceived and designed the experiments: JA, MM, SF. Performed the experiments and analyzed the data: JA. Bioinformatics analysis: DH. Wrote the paper: JA, SF.

### Conflict of interest statement

The authors declare that the research was conducted in the absence of any commercial or financial relationships that could be construed as a potential conflict of interest.

## Abbreviations

BSI: bloodstream infections
ESBL: Extended-spectrum beta-lactamase
MDR: multi-drug resistant
QRDR: quinolone resistance-determining region
UTI: urinary tract infection.
