# Supplementary material for: Exploring the Genome and Phenotype of Multi-Drug Resistant Klebsiella pneumoniae of Clinical Origin
Source: Front Microbiol. 2017 Oct 23;8:1913. doi: 10.3389/fmicb.2017.01913 (PMC5660112; doi:10.3389/fmicb.2017.01913)
Supplement: Supplementary file 1 [file DataSheet1.docx]

Supplementary Material

**Exploring the genome and phenotype of multi-drug resistant *Klebsiella pneumonia* of clinical origin**

João Anes, Daniel Hurley, Marta Martins and Séamus Fanning^§^

**Corresponding author:** Professor Séamus Fanning, [sfanning@ucd.ie](mailto:sfanning@ucd.ie)

## Supplementary material

## Supplementary Tables

**Table S1.** Overview of the assembly of eleven Klebsiella pneumoniae clinical isolates cultured from a tertiary teaching hospital in Dublin*.

| **Bacterial isolates**  **Features** | **CFS0363** | **CFS0364** | **CFS0365** | **CFS0366** | **CFS0367** | **CFS0368** | **CFS0369** | **CFS0370** | **CFS0371** | **CFS0372** | **CFS0373** |
| --- | --- | --- | --- | --- | --- | --- | --- | --- | --- | --- | --- |
| Accession number (SRR) | **5886493** | **5886494** | **5886491** | **5886492** | **5886497** | **5886498** | **5886495** | **5886496** | **5886499** | **5886500** | **5886490** |
| Number of contigs | 194 | 79 | 114 | 122 | 56 | 70 | 129 | 112 | 163 | 103 | 108 |
| Number of contigs (>= 1,000 bp) | 123 | 64 | 92 | 96 | 42 | 56 | 100 | 93 | 79 | 78 | 78 |
| Total Length (bp) | 5832316 | 5491098 | 5438843 | 5445420 | 5235101 | 5470012 | 5445048 | 5441494 | 5413888 | 5365262 | 5393903 |
| Total Length (>= 1,000 bp) | 5796883 | 5484851 | 5427545 | 5432430 | 5229603 | 5464492 | 5431374 | 5432066 | 5381288 | 5352263 | 5379074 |
| Largest contig (bp) | 763418 | 593388 | 361748 | 361463 | 539193 | 575504 | 359978 | 361747 | 361949 | 361463 | 361747 |
| GC (%) | 56.85 | 57.14 | 57.28 | 57.26 | 57.53 | 57.35 | 57.26 | 57.27 | 57.26 | 57.31 | 57.3 |
| N50^a^ | 224409 | 222905 | 197602 | 192863 | 262191 | 275033 | 170416 | 170518 | 167754 | 172807 | 167754 |
| N75^b^ | 130043 | 100571 | 89823 | 95597 | 179405 | 180991 | 89823 | 91153 | 91153 | 91153 | 91153 |
| L50^c^ | 8 | 9 | 11 | 11 | 8 | 7 | 12 | 11 | 12 | 11 | 12 |
| L75^d^ | 16 | 18 | 22 | 21 | 14 | 13 | 24 | 23 | 23 | 22 | 23 |
| Total CDS | 5417 | 5047 | 5099 | 5104 | 4836 | 5081 | 5100 | 5100 | 5055 | 5025 | 5049 |
| rRNA | 7 | 3 | 10 | 10 | 6 | 7 | 10 | 10 | 10 | 9 | 10 |
| tRNA | 83 | 82 | 81 | 85 | 81 | 82 | 82 | 82 | 82 | 76 | 84 |

^*^ Data generated by Quast 2.3 and PROKKA 1.1

^a^ N50 is defined as the length of the contig which, along with all the longer contigs, covers at least 50% of the whole genome.

^b^ N75 is defined as the length of the contig which, along with all the longer contigs, covers at least 75% of the whole genome.

^c^ L50 is defined as the lowest number of contigs required to cover at least 50% of the whole genome.

^d^ L75 is defined as the lowest number of contigs required to cover at least 75% of the whole genome.

**Table S2.** Genomic characterisation of antimicrobial-, virulence-, biocide- and metal-resistance encoding genes in Klebsiella pneumoniae study isolates extracted from the genome data using queried searches of selected databases.

| ***Klebsiella pneumoniae*** | **MLST** | **AMR resistance genes** | **Virulence associated genes** | **Biocide resistance genes** | **Metal resistance genes** |
| --- | --- | --- | --- | --- | --- |
| **CFS0363** | **ST147** | *aac(3)-IId, aac(6')-Ib, aadA1, ARR-3, bla*_CMY-4_*, bla*_CTX-M-15_*, bla*_OXA-1_*, bla*_OXA-9_*, bla*_SHV-12_*, bla*_TEM-1A_*, catA1, catB3, dfrA5, ere(A), ere(B), fosA, oqxA, oqxB, QnrB1, strA, strB, sul1, sul2, tet(A)* | ***entABES****,* ***fepABCDG****, fimABE,* ***fyuA****,* ***irp1****,* ***irp2****, mgtC, ompA, xcpR, ecpEDCBAR,* ***ybtAEPQSTUX****, mrkABCDFHIJ* | *cepA, emmdR, fabI, kpnEF, kexD, kpnO, kmrA,* *qacEΔ1, sugE* | *arsBCR, corABCD, cusCBA, mdtABC-tolC, merACDEPT, modABCE, nikBCR, pcoABCDRSE, pstSACB, silABCDEFPRS, sitABCD, terABCDEZ, znuABC* |
| **CFS0364** | **ST15** | *aac(3)-IIa, bla*_CTX-M-15_*, bla*_SHV-28_*, bla*_TEM-1B_*, fosA* | ***entABES****,* ***fepABCDG****, fimABCE,* ***fyuA****,* ***irp1****,* ***irp2****, mgtC, ompA, xcpR, ecpEDCBAR,* ***ybtAEPQSTUX****, mrkABCDFHIJ,* ***kfuABC*** | *cepA, emmdR, fabI, kpnEF, kpnO, kmrA, sugE* | *arsABCDHR, corABCD, cusCBA, mdtABC-tolC, merACDEPT, modABCE, nikBCR, pcoABCDRSE, pstSACB, silABCDEFPRS, sitABCD, znuABC* |
| **CFS0365** | **ST340** | *aac(3)-IId, aadA2, bla*_CTX-M-15_*, bla*_SHV-12_*, bla*_TEM-1B_*, catA2, dfrA12, dfrA14, fosA, oqxA, oqxB, strA, strB, sul1, sul2, tet(D)* | ***entABES****,* ***fepABCDG****, fimAE, mgtC, ompA, xcpR, ecpEDCBAR, mrkABCDFHIJ* | *cepA, emmdR, fabI, kpnEF, kexD, kpnO, kmrA, qacEΔ1, sugE* | *arsABCDHR, corABCD, cusCBA, mdtABC-tolC, merACDEPT, modABCE, nikBCR, pcoABCDRSE, pstSACB, silABCDEFPRS, sitABCD, znuABC* |
| **CFS0366** | **ST340** | *aac(3)-IId, aadA2, bla*_CTX-M-15_*, bla*_SHV-12_*, bla*_TEM-1B_*, catA2, dfrA12, dfrA14, fosA, oqxA, oqxB, strA, strB, sul1, sul2, tet(D)* | ***entABES****,* ***fepABCDG****, fimAE, mgtC, ompA, xcpR, ecpEDCBAR, mrkABCDFHIJ* | *cepA, emmdR, fabI, kpnEF, kexD, kpnO, kmrA, qacEΔ1, sugE* | *arsABCDHR, corABCD, cusCBA, mdtABC-tolC, merACDEPT, modABCE, nikBCR, pcoABCDRSE, pstSACB,silABCDEFPRS, sitABCD, znuABC* |
| **CFS0367** | **ST14** | *bla*_ACC-1_*, bla*_SHV-28_*, fosA, oqxA, oqxB* | ***entABES****,* ***fepABCDG****, fimABE, mgtC, ompA, xcpR, ecpEDCBAR, mrkABCDFHIJ,* ***kfuABC*** | *cepA, emmdR, fabI, kpnEF, kpnO, kmrA, sugE* | *arsBHR, corABCD, cusCBA, mdtABC-tolC, modABCE, nikBCR, pstSACB, sitABCD, znuABC* |
| **CFS0368** | **ST14** | *bla*_ACC-1_*, bla*_SHV-28_*, dfrA16, fosA, oqxA, oqxB, sul1* | ***entABES****,* ***fepABCDG****, fimABE, mgtC, ompA, xcpR, ecpEDCBAR, mrkABCDFHIJ,* ***kfuABC*** | *cepA, emmdR, fabI, kpnEF, kpnO, kmrA, qacEΔ1, sugE* | *arsABCDHR, corABCD, mdtABC-tolC, cusCBA, merACDEFPT, modABCE, nikBCR, pcoABCDRSE, pstSACB, silABCDEFPRS, sitABCD, znuABC* |
| **CFS0369** | **ST340** | *aac(3)-IId, bla*_CTX-M-15_*, bla*_SHV-12_*, bla*_TEM-1B_*, catA2, dfrA12, dfrA14, fosA, oqxA, oqxB, strA, strB, sul1, sul2, tet(D)* | ***entABES****,* ***fepABCDG****, fimAE, mgtC, ompA, xcpR, ecpEDCBAR, mrkABCDFHIJ* | *cepA, emmdR, fabI, kpnEF, kexD, kpnO, kmrA, qacEΔ1, sugE* | *arsABCDHR, corABCD, cusCBA, mdtABC-tolC, merACDEPT, modABCE, nikBCR, pcoABCDRSE, pstSACB, silABCDEFPRS, sitABCD, znuABC* |
| **CFS0370** | **ST340** | *aac(3)-IId, aadA2, bla*_CTX-M-15_*, bla*_SHV-12_*, bla*_TEM-1B_*, catA2, dfrA12, dfrA14, fosA, oqxA, oqxB, strA, strB, sul1, sul2, tet(D)* | ***entABES****,* ***fepABCDG****, fimAE, mgtC, ompA, xcpR, ecpEDCBAR,* *mrkABCDFHIJ* | *cepA, emmdR, fabI, kpnEF, kexD, kpnO, kmrA, qacEΔ1, sugE* | *arsABCDHR, corABCD, cusCBA, mdtABC-tolC, merACDEPT, modABCE, nikBCR, pcoABCDRSE, pstSACB, silABCDEFPRS, sitABCD, znuABC* |
| **CFS0371** | **ST340** | *aadA2, bla*_CTX-M-15_*, bla*_SHV-11_*, dfrA12, dfrA14, fosA, oqxA, oqxB, sul1* | ***entABES****,* ***fepABCDG****, fimAE, mgtC, ompA, xcpR, ecpEDCBAR, mrkABCDFHIJ* | *cepA, emmdR, fabI, kpnEF, kexD,kpnO, kmrA, qacEΔ1, sugE* | *arsABCDHR, corABCD, cusCBA, mdtABC-TolC, merACDEPT, modABCE, nikBCR, pcoABCDRSE, pstSACB, silABCDEFPRS, sitABCD, znuABC* |
| **CFS0372** | **ST340** | *aadA2, bla*_CTX-M-15_*, bla*_SHV-12_*, dfrA14, fosA, oqxA, oqxB, sul1* | ***entABES****,* ***fepABCDG****, fimAE, mgtC, ompA, xcpR, ecpEDCBAR, mrkABCDFHIJ* | *cepA, emmdR, fabI, kpnEF, kexD, kpnO, kmrA, qacEΔ1, sugE* | *arsABCDHR, corABCD, cusCBA, mdtABC-tolC, merACDEPT, modABCE, nikBCR, pcoABCDRSE, pstSACB, silABCDEFPRS, sitABCD, znuABC* |
| **CFS0373** | **ST340** | *aadA2, bla*_CTX-M-15_*, bla*_SHV-11_*, dfrA12, dfrA14, fosA, oqxA, oqxB, sul1* | ***entABES****,* ***fepABCDG****, fimAE, mgtC, ompA,xcpR, ecpEDCBAR, mrkABCDFHIJ* | *cepA, emmdR, fabI, kpnEF, kexD, kpnO, kmrA, qacEΔ1, sugE* | *arsABCDHR, corABCD, cusCBA, mdtABC-tolC, merACDEPT, modABCE, nikBCR, pcoABCDRSE, pstSACB, silABCDEFPRS, sitABCD, znuABC* |

**Table S3.** Amino acid substitutions identified in various protein encoding genes in Klebsiella pneumonia of clinical origin. The proteins were clustered based on their function and isolates were compared against isolates of the same Sequence Type (ST). Grey shading – absence of mutations; Trunc – protein truncated; NP – not present.

| **Function** | **Protein** | **CG147** | **CG15** | | | **CG258** | | | | | | |
| --- | --- | --- | --- | --- | --- | --- | --- | --- | --- | --- | --- | --- |
|  |  | **CFS0363** | **CFS0364** | **CFS0367** | **CFS0368** | **CFS0365** | **CFS0366** | **CFS0369** | **CFS0370** | **CFS0371** | **CFS0372** | **CFS0373** |
| **Efflux** | **AcrA** |  |  |  |  |  |  |  |  |  |  |  |
|  | **AcrB** |  |  |  | R715L |  |  |  |  |  |  |  |
|  | **AcrR** |  |  | Trunc |  |  |  |  |  |  |  |  |
|  | **TolC** |  |  |  |  |  |  |  |  |  |  |  |
| **Global regulators** | **MarA** |  |  |  |  |  |  |  |  |  |  |  |
|  | **MarB** |  |  |  |  |  |  |  |  |  |  |  |
|  | **MarR** |  |  |  |  |  |  |  |  |  |  |  |
|  | **RamA** |  | NP | NP |  |  |  |  |  |  |  |  |
|  | **RamR** | K194- | NP | NP | K194- |  |  |  |  |  |  |  |
|  | **Rob** |  |  |  |  |  |  |  |  |  |  |  |
|  | **SoxR** |  |  |  |  |  |  |  |  |  |  |  |
|  | **SoxS** |  |  |  |  |  |  |  |  |  |  |  |
| **OM** | **OmpK36** |  |  | D135- G136-K188N | D135- G136-K188N |  |  |  |  |  |  |  |
|  | **OmpK35** |  |  |  |  |  |  |  |  |  |  |  |
| **QRDR** | **GyrA** |  | N645H | F83Y G87D E48- | F83Y G87D E48- |  |  |  |  |  |  |  |
|  | **GyrB** |  |  | E466D |  |  |  |  |  |  |  |  |
|  | **ParC** |  |  | G84E | G84E |  |  |  |  |  |  |  |
|  | **ParE** |  |  | Q460P |  |  |  |  |  |  |  |  |
| **Biofilm** | **UlaA** |  |  |  | Y268H |  |  |  |  |  |  |  |
|  | **UlaB** |  |  |  |  |  |  |  |  |  |  |  |
|  | **UlaC** |  |  |  |  |  |  |  |  |  |  |  |
|  | **YfiB** |  |  | G79A G80A | G79A G80A |  |  |  |  |  |  |  |
|  | **YfiN** |  |  | M270K |  |  |  |  |  |  |  |  |
|  | **YfiR** |  |  |  |  |  |  |  |  |  |  |  |
|  | **AstA** |  |  |  |  |  |  |  |  |  |  |  |
|  | **AstB** |  |  |  |  |  |  |  |  |  |  |  |
|  | **AstC** |  |  |  | A176V |  |  |  |  |  |  |  |
|  | **AstD** |  |  | A74V |  |  |  |  |  |  |  |  |
|  | **FrdA** |  |  |  |  |  |  |  |  |  |  |  |
|  | **FrdB** |  |  |  |  |  |  |  |  |  |  |  |
|  | **FrdC** |  |  |  |  |  |  |  |  |  |  |  |
|  | **FrdD** |  |  |  |  |  |  |  |  |  |  |  |
|  | **OmpR** |  |  |  |  |  |  |  |  |  |  |  |
|  | **OmpW** |  |  |  |  |  |  |  |  |  |  |  |
| **fimbriae and cellulose** | **BcsA** |  |  |  |  |  |  |  |  |  |  |  |
|  | **BcsB** |  |  |  |  |  |  |  |  |  |  |  |
|  | **BcsC** |  |  |  |  |  |  |  |  |  |  |  |
|  | **BcsZ** |  |  |  |  |  |  |  |  |  |  |  |
|  | **Crl/CsgA** |  |  |  |  |  |  |  |  |  |  |  |
|  | **MrkA** |  |  |  |  |  |  |  |  |  |  |  |
|  | **CsgD** |  |  |  |  |  |  |  |  |  |  |  |

## Supplementary Figures

**Figure S1.** Biofilm formation for Klebsiella pneumoniae clinical isolates grown statically in Müeller-Hinton broth at 25˚C for 96 hours.

**Figure S2.** Biofilm formation for Klebsiella pneumoniae clinical isolates grown statically in Müeller-Hinton broth at 37˚C for 96 hours.

**Figure S3.** Biofilm formation for Klebsiella pneumoniae clinical isolates grown statically in M9 minimal broth at 25˚C for 96 hours.

**Figure S4.** Biofilm formation for Klebsiella pneumoniae clinical isolates grown statically in M9 minimal broth at 37˚C for 96 hours.


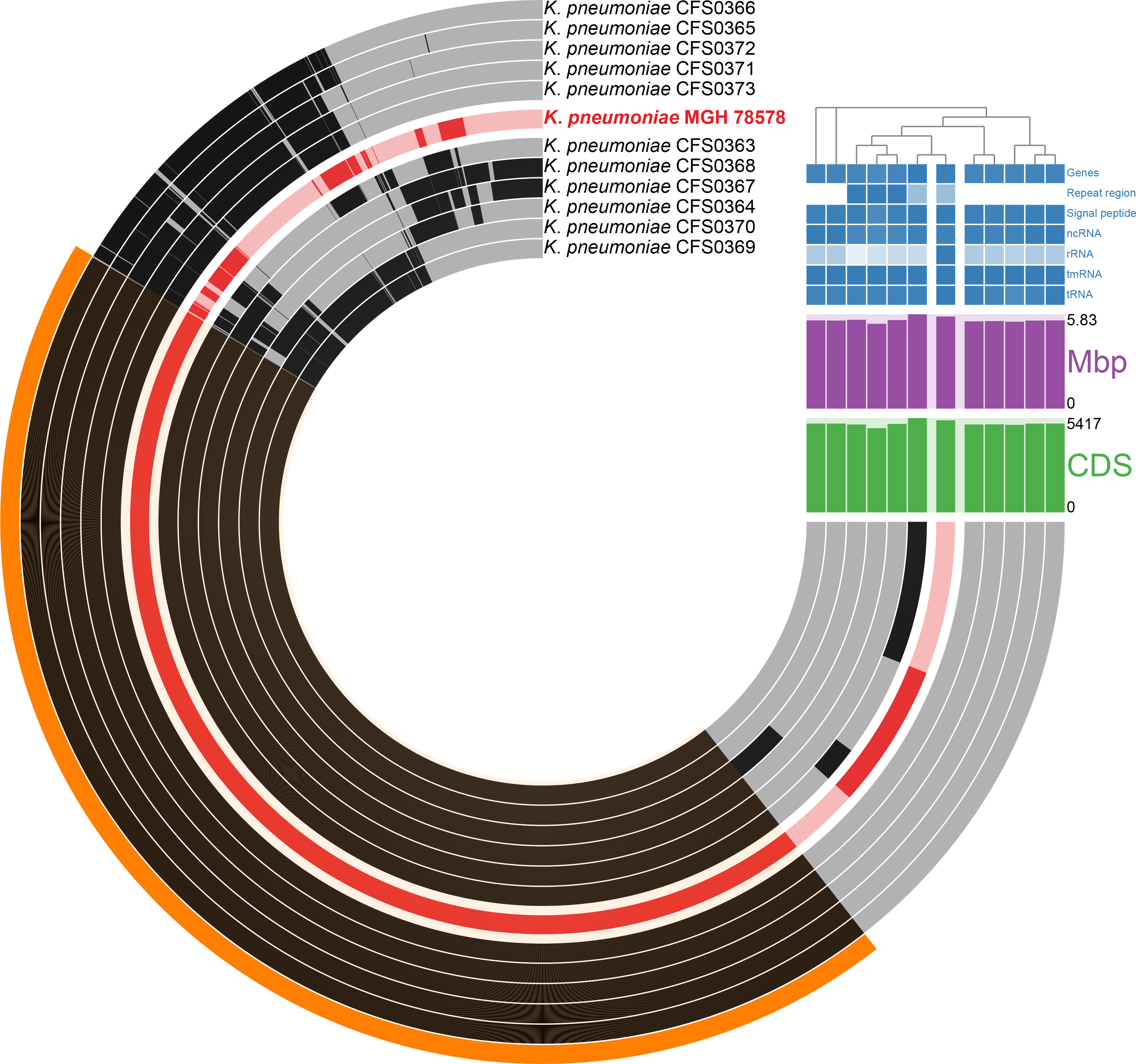


**Figure S5.** An Anvi’o representation of the pan-genome for all 11 *Klebsiella pneumoniae*. The genomes are clustered based on the presence/absence pattern of protein clusters indicated in black, the absence of protein cluster is indicated in grey. The outer ring shown in orange represents the core genome of *Klebsiella pneumoniae* when compared with *K. pneumoniae* MGH 78578 marked in red. The blocks shown in green represent the number of coding sequences (CDS) in each genome; sizes of each of genome, in Mbp is indicated in purple and sizes in each case; phylogenetic tree showing the relationship among the clinical isolates is indicated in blue.

MALDI-TOF spectra


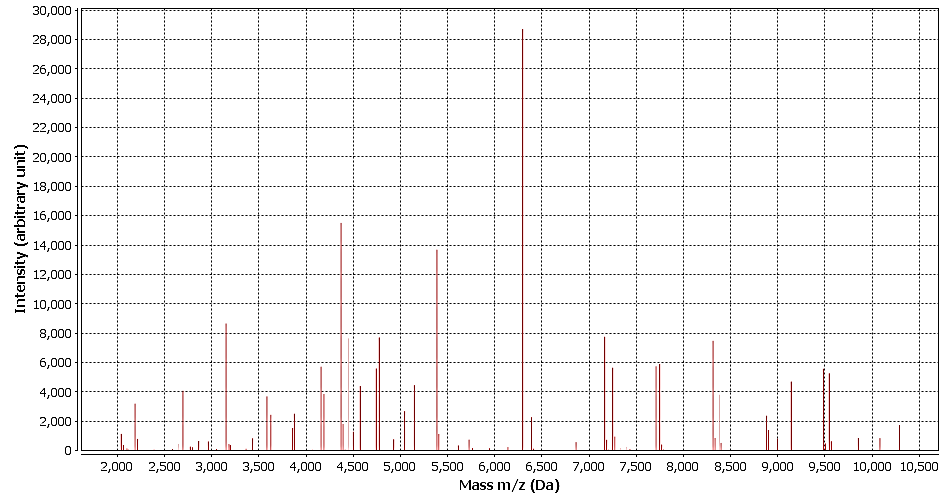


**Figure S6.1.** MALDI-TOF mass spectrum obtained for Klebsiella pneumoniae isolate CFS0363 following growth in Müeller-Hinton Agar.


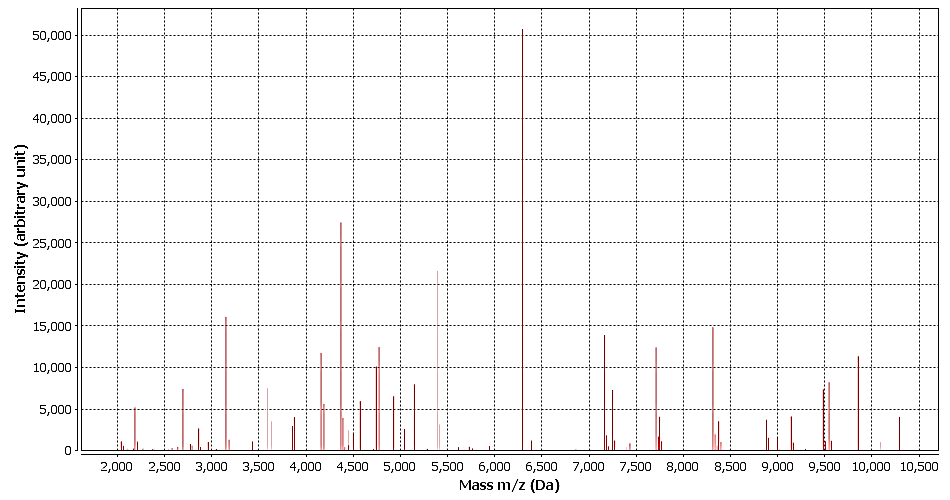


**Figure S6.2.** MALDI-TOF mass spectrum obtained for Klebsiella pneumoniae isolate CFS0364 following growth in Müeller-Hinton Agar.


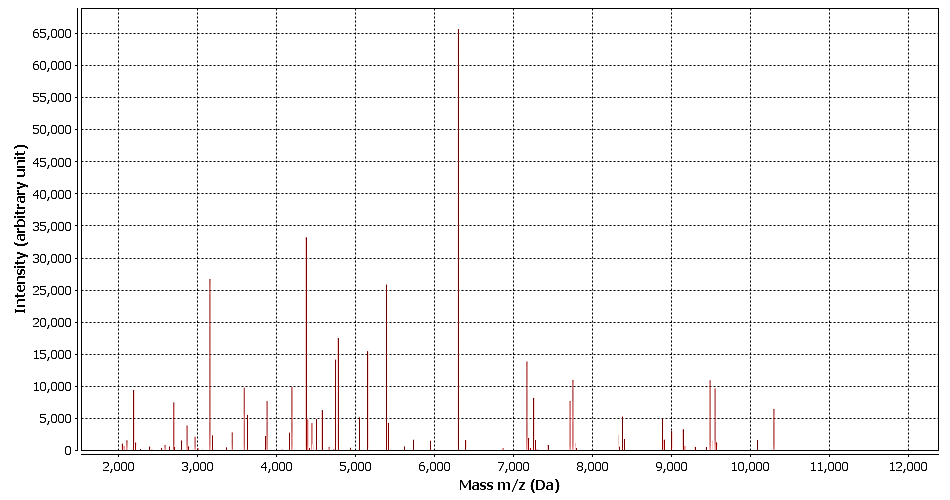


**Figure S6.3.** MALDI-TOF mass spectrum obtained for Klebsiella pneumoniae isolate CFS0365 following growth in Müeller-Hinton Agar.


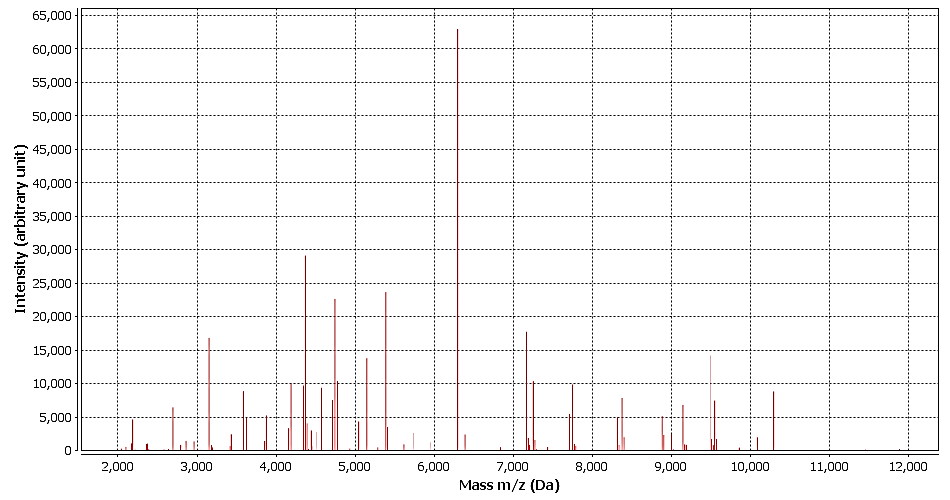


**Figure S6.4.** MALDI-TOF mass spectrum obtained for Klebsiella pneumoniae isolate CFS0366 following growth in Müeller-Hinton Agar.


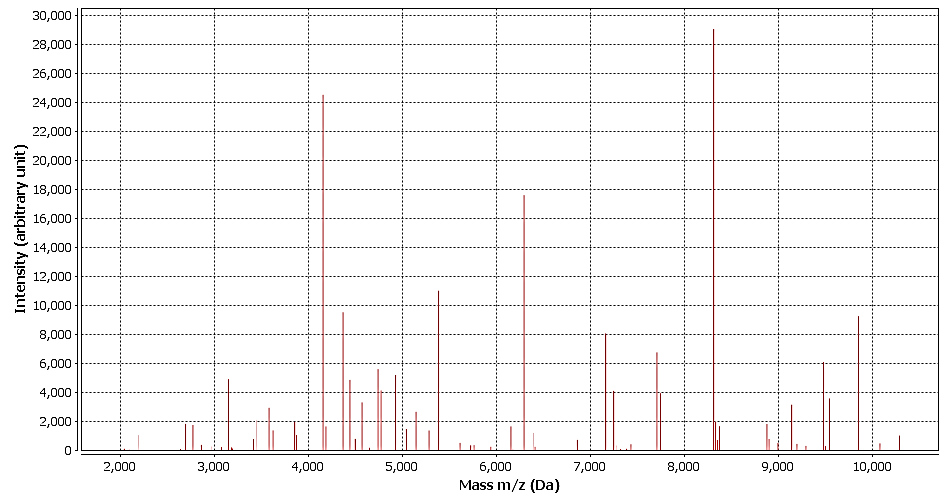


**Figure S6.5.** MALDI-TOF mass spectrum obtained for Klebsiella pneumoniae isolate CFS0367 following growth in Müeller-Hinton Agar.


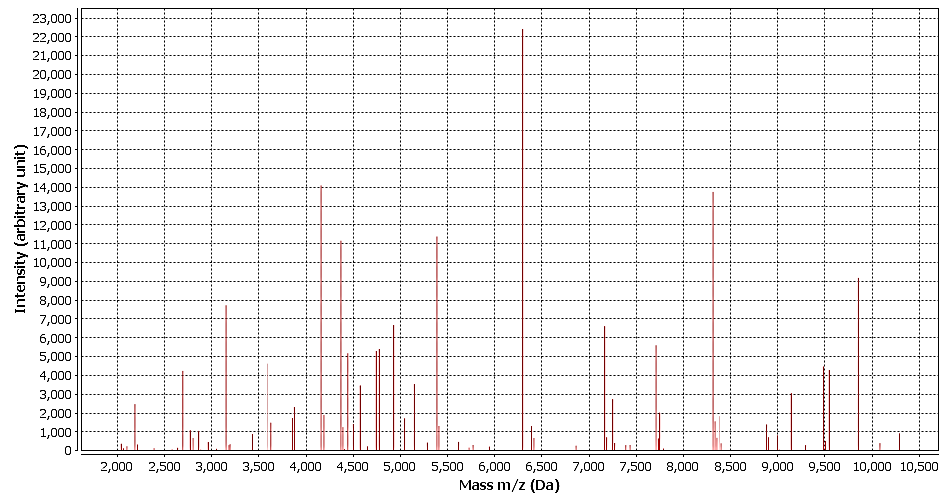


**Figure S6.6.** MALDI-TOF mass spectrum obtained for Klebsiella pneumoniae isolate CFS0368 following growth in Müeller-Hinton Agar.


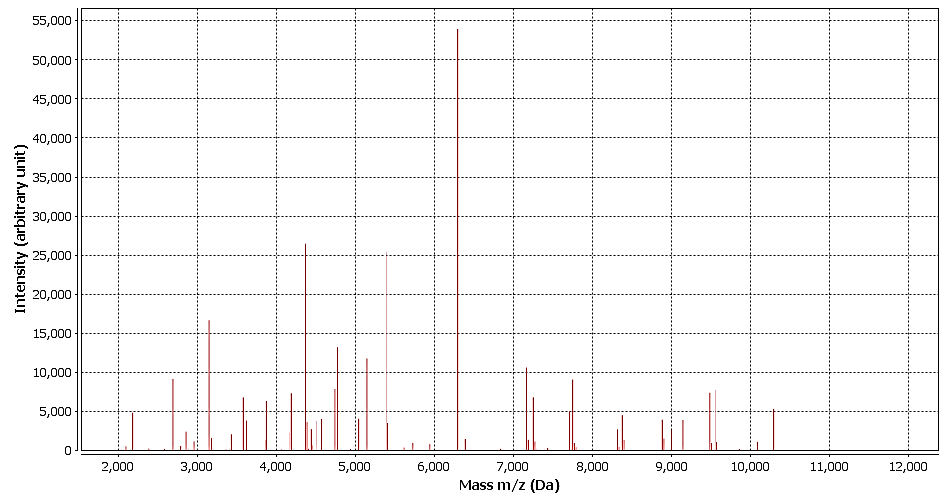


**Figure S6.7.** MALDI-TOF mass spectrum obtained for Klebsiella pneumoniae isolate CFS0369 following growth in Müeller-Hinton Agar.


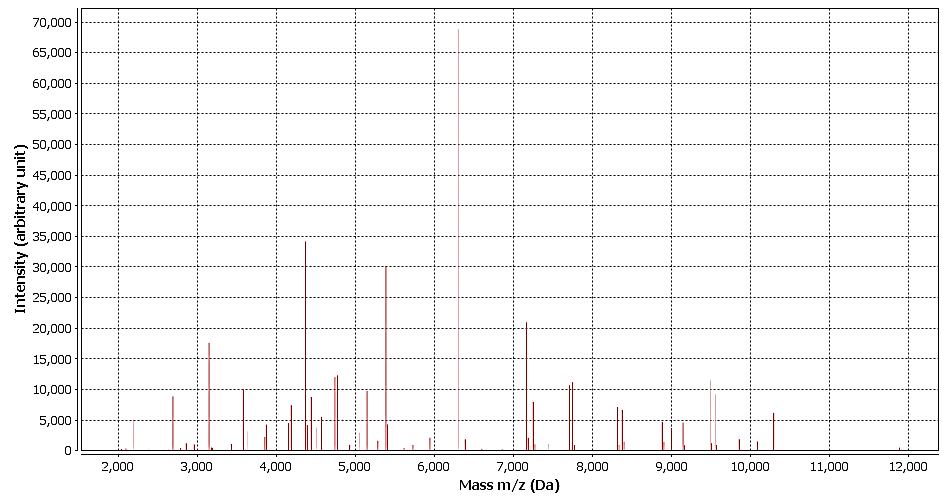


**Figure S6.8.** MALDI-TOF mass spectrum obtained for Klebsiella pneumoniae isolate CFS0370 following growth in Müeller-Hinton Agar.


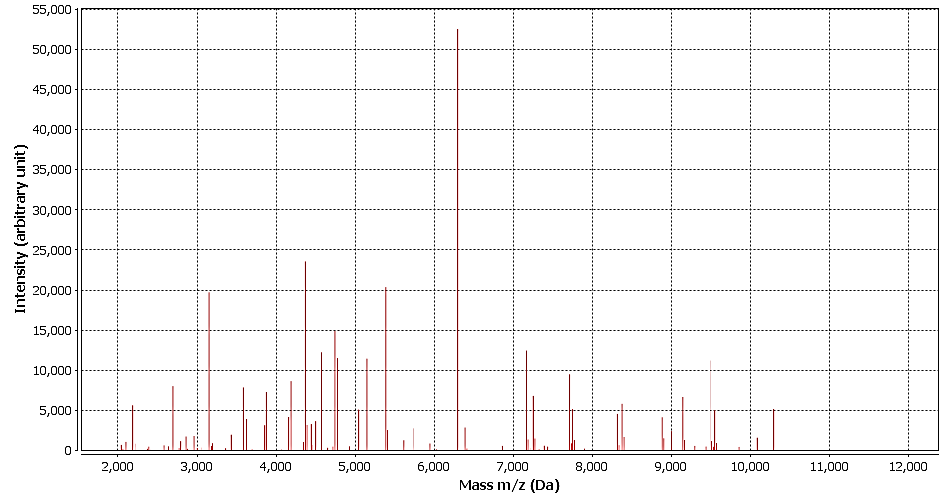


**Figure S6.9.** MALDI-TOF mass spectrum obtained for Klebsiella pneumoniae isolate CFS0371 following growth in Müeller-Hinton Agar.


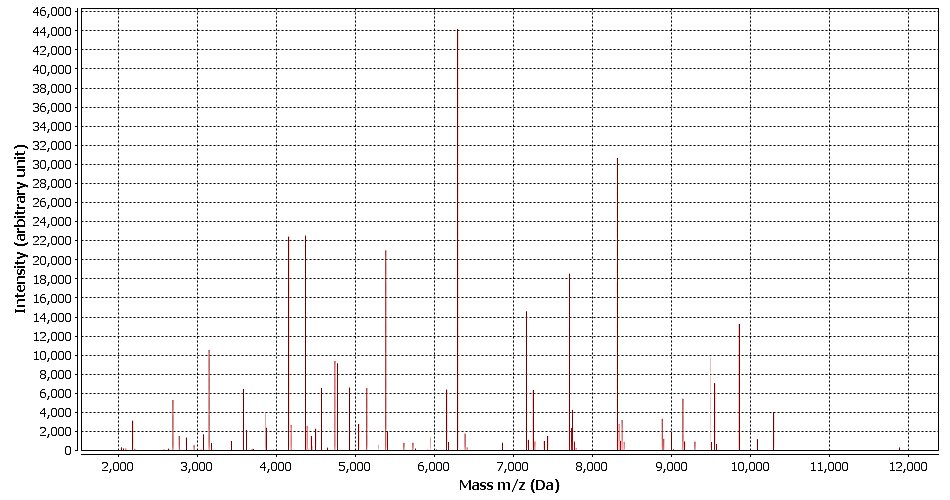


**Figure S6.10.** MALDI-TOF mass spectrum obtained for Klebsiella pneumoniae isolate CFS0372 following growth in Müeller-Hinton Agar.


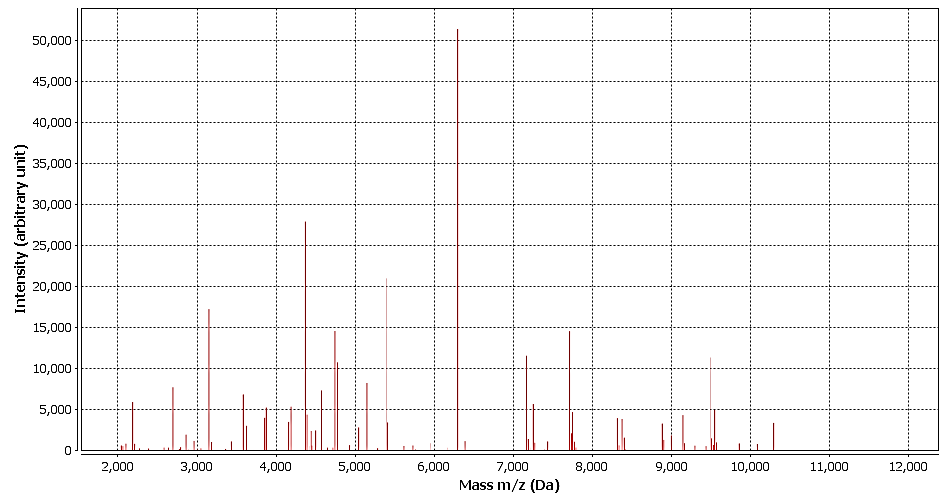


**Figure S6.11.** MALDI-TOF mass spectrum obtained for Klebsiella pneumoniae isolate CFS0373 following growth in Müeller-Hinton Agar.
